# Deleterious effects of endocrine disruptors are corrected in the mammalian germline by epigenome reprogramming

**DOI:** 10.1186/s13059-015-0619-z

**Published:** 2015-03-27

**Authors:** Khursheed Iqbal, Diana A Tran, Arthur X Li, Charles Warden, Angela Y Bai, Purnima Singh, Xiwei Wu, Gerd P Pfeifer, Piroska E Szabó

**Affiliations:** Department of Molecular and Cellular Biology, Beckman Research Institute, City of Hope, Duarte, California 91010 USA; Irell and Manella Graduate School of Biological Sciences, City of Hope, Duarte, California 91010 USA; Department of Information Science, Beckman Research Institute, City of Hope, Duarte, California 91010 USA; Eugene and Ruth Roberts Summer Academy, City of Hope, Duarte, California 91010 USA; Department of Cancer Biology, Beckman Research Institute, City of Hope, Duarte, California 91010 USA; Center for Epigenetics, Van Andel Research Institute, Grand Rapids, Michigan 49503 USA

**Keywords:** endocrine disruptor, germline epigenetic reprogramming, DNA methylation, vinclozolin, bisphenol A, DEHP, transgenerational epigenetic inheritance, fetal germ cell, genomic imprinting

## Abstract

**Background:**

Exposure to environmental endocrine-disrupting chemicals during pregnancy reportedly causes transgenerationally inherited reproductive defects. We hypothesized that to affect the grandchild, endocrine-disrupting chemicals must alter the epigenome of the germ cells of the *in utero*-exposed G1 male fetus. Additionally, to affect the great-grandchild, the aberration must persist in the germ cells of the unexposed G2 grandchild.

**Results:**

Here, we treat gestating female mice with vinclozolin, bisphenol A, or di-(2-ethylhexyl)phthalate during the time when global *de novo* DNA methylation and imprint establishment occurs in the germ cells of the G1 male fetus. We map genome-wide features in purified G1 and G2 prospermatogonia, in order to detect immediate and persistent epigenetic aberrations, respectively. We detect changes in transcription and methylation in the G1 germline immediately after endocrine-disrupting chemicals exposure, but changes do not persist into the G2 germline. Additional analysis of genomic imprints shows no persistent aberrations in DNA methylation at the differentially methylated regions of imprinted genes between the G1 and G2 prospermatogonia, or in the allele-specific transcription of imprinted genes between the G2 and G3 soma.

**Conclusions:**

Our results suggest that endocrine-disrupting chemicals exert direct epigenetic effects in exposed fetal germ cells, which are corrected by reprogramming events in the next generation. Avoiding transgenerational inheritance of environmentally-caused epigenetic aberrations may have played an evolutionary role in the development of dual waves of global epigenome reprogramming in mammals.

**Electronic supplementary material:**

The online version of this article (doi:10.1186/s13059-015-0619-z) contains supplementary material, which is available to authorized users.

## Background

Humans are broadly exposed to synthetic endocrine-disrupting chemicals (EDs) from the environment [1-5]. EDs closely resemble endogenous hormones in structure and have been reported to cause developmental and reproductive health problems [6-10]. EDs have the ability to affect gene expression and DNA methylation [11]. It has been suggested that one initial exposure to EDs *in utero* harms multiple generations in rodents and that the underlying mechanism is epigenetic [6,12-14]. However, the molecular mechanisms mediating such ED exposure-dependent transgenerational inheritance in the germline have not been identified.

**Figure 1:**
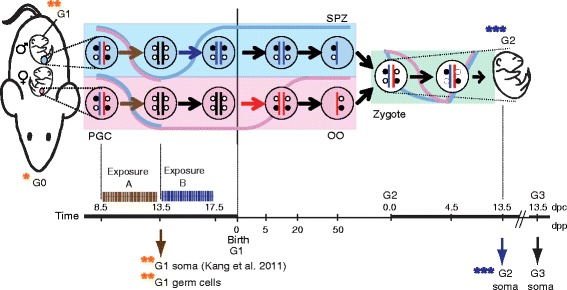
**Assessing ED effects on imprint reprogramming in the mouse germline. (Top)** Reprogramming in the mouse germline. Exposure of pregnant mice to EDs may directly affect the G0 dam and the G1 soma and G1 germline, and indirectly, the G2 soma that develops from G1 germ cells. DNA methylation patterns are globally remodeled during gametogenesis in both male (above) and female (below) embryos/fetuses in the chromosomes that are paternally (blue) or maternally (red) inherited. The 5-methylcytosine levels (blue and pink curves) are globally reduced in the primordial germ cells (PGC) of both sexes; DNA methylation of imprinted DMRs is similarly erased (brown horizontal arrow) by mid-gestation (13.5 dpc). In the male germ cells, global DNA methylation is largely reset and paternal imprints are newly established (blue arrow) in prospermatogonia prior to birth and maintained (black horizontal arrows) into spermatozoa (SPZ). In female germ cells, global DNA methylation and maternal imprints are established (red arrow) after birth during oocyte (OO) growth (from 5 to 20 dpp). The imprinting marks are maintained (black arrows) through global remodeling at fertilization and embryo development. **(Bottom)** Daily gavage was given to pregnant dams in the time windows of ‘Exposure A’ or ‘Exposure B’ to affect the erasure phase of the germline reprogramming in PGCs or the establishment phase in MGC. The timeline (not to scale) is marked with gestational age (dpc) on top, and also with the age after birth (dpp) at the bottom. In our related publication [32], maintenance of imprinting was analyzed in the exposed G1 soma after ‘Exposure A’. In the current study, we focused on the effect of EDs on imprinting via the exposed G1 germ cells. Experimental endpoints are indicated by the arrows pointing down.

*In utero* ED exposure may harm epigenetic remodeling events (Figure 1) in the germline of the embryo or fetus [15]. In the mouse, such events include global erasure of DNA methylation in primordial germ cells (PGCs) in embryos of both sexes [16-18] and *de novo* establishment of the sperm-type DNA methylation in fetal male germ cells (MGCs) [19]. *De novo* DNA methylation in the female germline may be less vulnerable to *in utero* exposures, as it takes place after birth in the growing oocytes of the juvenile female [20,21].

An important part of the germline remodeling process is the resetting of genomic imprints (Figure 1). Imprinted genes control important developmental processes, including pre- and postnatal growth, metabolism, and behavior. Failure to reprogram imprinted genes in the germ line due to a compromised *in utero* environment is of special concern [22-24]. Parental allele-specific monoallelic transcription of imprinted genes in the soma mainly depends on the resetting - erasure and re-establishment - of their differentially methylated regions (DMRs) in the male and female germlines [25]. DNA methylation marks at DMRs are erased in PGCs in both sexes by 13.5 days post coitum (dpc) (Figure 1) [26,27]. Erasure of imprints results in a shift from monoallelic to biallelic expression of imprinted genes [28,29]. Re-establishment of the male-specific DNA methylation marks occurs at paternally methylated (PAT) DMRs in prospermatogonia and this methylation is thereafter maintained to the spermatozoa stage [30]. Whereas PAT DMRs appear to follow the default global pattern of *de novo* DNA methylation in prospermatogonia, MAT DMRs in the same cells are protected from *de novo* DNA methylation at sites of H3K4 methylation [19]. Sperm- or oocyte-specific imprinting marks continue to be maintained following fertilization during the global wave of epigenetic remodeling that takes place in the zygote and early embryo [31] and are further maintained in the soma in the paternally and maternally inherited chromosomes.

Several studies have reported that the process of genomic imprinting is perturbed by endocrine disruptors. The maintenance of imprinting in the in utero exposed embryo is largely resistant but not completely immune to the effects of EDs, as can be seen from the minor aberrations and increased noise in allele-specific transcription of imprinted genes [32]. PGCs at 12.5 dpc exhibit an accelerated imprint erasure rate at the *Igf2r*, *Peg3*, and *H19* DMRs after *in utero* BPA exposure [33]. *In utero* exposure to VZ led to decreased DNA methylation of PAT DMRs in mouse spermatozoa [6,34,35] in the exposed and further generations, suggesting that the establishment and erasure steps were both disturbed. The establishment step of maternal imprints was affected in mouse oocytes: BPA administration to juvenile females caused a reduction in CpG methylation at the *Igf2r-* and *Peg3* MAT DMRs [36]. The maintenance step of genomic imprinting was perturbed in the offspring after BPA exposure of mothers shortly before and during pregnancy [37]. Human severe azoospermia is associated with DNA methylation imprint defects at the *H19-IGF2* DMR [38,39], but it is not known whether EDs have a role in these aberrations.

We hypothesized that any epigenetic aberration causing transgenerationally inherited phenotype in G2 and G3 individuals after *in utero* exposure of the male G1 germline must meet the following criteria: (1) it is present in the fetal G1 germ cell immediately after exposure; (2) it persists into the G1 gamete; and (3) it persists into the unexposed G2 fetal germ cell of the next generation. In this study, we sought to detect such immediate and persistent aberrations. We analyzed *in utero*-exposed G1 prospermatogonia and unexposed G2 prospermatogonia to detect immediate and persistent effects of EDs on global transcription. We searched for immediate and persistent changes in DNA methylation in G1 and G2 prospermatogonia and also in G1 and G2 adult spermatozoa. To further analyze the effects of EDs on the imprinting process we also followed the potential germline epigenetic aberration into the G2 and G3 soma. Our data collectively show that the male germline suffers from immediate epigenetic effects after *in utero* BPA, DEHP, and VZ exposure but recovers in the subsequent generation.

## Results

The aim of this study was to systematically and rigorously evaluate the effects of EDs on global epigenetic reprogramming and imprint resetting in the male germline after *in utero* exposure. We chose three EDs: vinclozolin(VZ) at 100 mg/kg/day; di-(2-ethylhexyl) phthalate (DEHP) at 750 mg/kg/day; and bisphenol A (BPA) at 0.2 mg/kg/day. These *in utero* doses have been reported to cause morphological or physiological defects, including reproductive harm in the G1 fetuses (see Methods section).

### Testing the effect of EDs on imprint erasure

**Figure 2:**
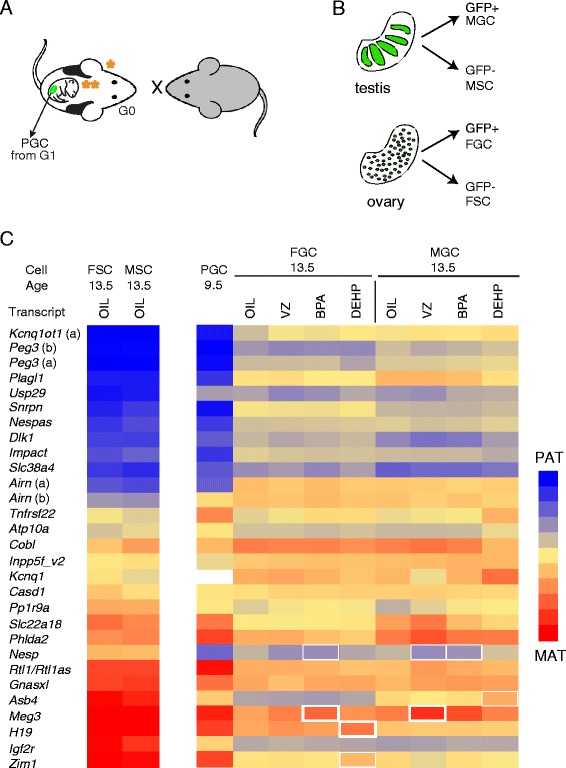
**Testing the effect of endocrine-disrupting chemicals (EDs) on imprint erasure. (A)** Experimental design. JF1 females (tail up) were mated with OG2 males (tail down). Pregnant dams (G0) were treated with EDs or oil control from 8.5 dpc to 12.5 dpc. Imprint erasure occurs during this time in PGCs of the exposed G1 embryos. One orange star = exposed G0 generation; two orange stars = exposed G1 embryo. **(B)** GFP positive male and female germ cells and somatic cells were collected from the gonads by FACS sorting. **(C)** Results of RNA Sequenom allelotyping experiments of imprinted transcripts are displayed for cells treated with an ED or control vehicle (‘oil’); letters in parentheses indicate separate SNPs. Average (n = 3) allele-specific transcription is shown in color, 100% paternal (blue) to 100% maternal (red), with 50% biallelic in yellow. Many imprinted genes have parental allele-specific transcription in PGCs at 9.5 dpc but biallelic transcription in FGCs and MGCs (but not in FSCs and MSCs) at 13.5 dpc. Statistically significant (*P* <0.05) aberrations in erased allele-specific transcription, greater than 5% or 10%, are marked by thin or bold white rectangles, respectively.

We treated pregnant dams daily from 8.5 dpc to 12.5 dpc (exposure A in Figure 1) with one of the EDs or with corn oil as control. For this experiment we used JF1 females and OG2 males (Figure 2A). The OG2 transgenic line carries an Oct4 promoter-GFP transgene that allowed us to purify the GFP-positive male and female germ cells (MGCs and FGCs) and GFP-negative somatic cells (MSCs and FSCs) from the dissected embryonic gonads by FACS sorting (Figure 2B). The JF1 inbred mouse strain is genetically distinct from the OG2 line, providing single nucleotide polymorphisms. This allowed measurement of the allele-specific transcription of known imprinted genes in JF1 × OG2 cells using multiplex RNA-single nucleotide primer extension (SNuPE) assays uisng Sequenom allelotyping [32].

We observed correct parental allele-specific transcription in control-treated FSCs and MSCs at 13.5 dpc for ubiquitously imprinted transcripts; for example, *Kcnq1ot1*, *Peg3*, *Plagl1*, and *Dlk1* were expressed from the paternal OG2 allele, and *Igf2r*, *H19*, and *Meg3* were expressed from the maternal JF1 allele (Figure 2C and Additional file 1). These expression patterns represent the normal correct ubiquitous parental allele-specific transcription. Some other transcripts (*Kcnq1*, *Phlda2*, and *Atp10a*, for example, which have tissue-specific or developmental stage-specific imprinting) displayed biallelic transcription in FSCs and MSCs. The parental allele-specific transcription in PGCs at 9.5 dpc switched to biallelic transcription in control FGCs and MGCs at 13.5 dpc, consistent with the erasure of genomic imprints.

Looking at the erasure of allele-specific transcription in ED-treated FGCs and MGCs, we found few aberrations that were greater than 5% and were statistically significant (Student’s *t*-test, *P* <0.05) (Table 1). *Meg3* was affected by BPA in FGCs and by VZ in MGCs; *H19* was affected by DEHP in FGCs; and *Zim1* was affected by DEHP in FGCs. Considering the number of tests (28 SNPs tested), we could expect one or two positive results for each ED in each cell type simply by chance (*P* <0.05); the number we found was close to that expectation. *Nesp* and *Asb4* appeared to have a change, but these genes did not exhibit correct parental allele-specific transcription in PGCs (Figure 2C). Therefore, these cannot be considered as erasure defects. Using multiple testing, we found that none of the erasure defects were significant for any of the EDs using the Bonferroni corrected *P* value (*P* <0.05/28 = 0.0018).

**Table 1:** **Significant changes between control and ED-treated samples after disturbing G1 germ line imprint erasure - RNA**

							**% MAT allele Avg. (n = 3)**	
**Transcript**	**Cell type**	**ED**	**Difference (%)**	**SD (%)**	**Student’s** ***t*** **-test** ***P*** **value**	**Imprinted base line (cutoff 80%)**	**Oil**	**Treatment**
*Nesp*	MGC	VZ	-9.1	3.440	0.004	NO (erased)	39	30
*Nesp*	FGC	BPA	-8.2	4.356	0.042	NO (erased)	38	30
*Nesp*	MGC	BPA	-7.9	0.956	0.001	NO (erased)	39	31
*Asb4*	MGC	DEHP	8.7	3.769	0.049	NO (erased)	54	63
*Zim1*	FGC	DEHP	9.0	5.406	0.030	NO (erased)	52	61
*H19*	FGC	DEHP	10.6	7.507	0.024	NO (erased)	65	75
*Meg3*	FGC	BPA	13.7	4.713	0.016	NO (erased)	65	79
*Meg3*	MGC	VZ	16.8	4.438	0.002	NO (erased)	73	90

Allele-specific transcription was compared between ED- and vehicle-treated samples for each transcript in 13.5 dpc FGC and MGC using SNuPE assays. Changes in the average (n = 3) allele-specific transcription that were greater than 5% and were statistically significant (*P* <0.05) were tabulated and ordered according to the difference in maternal allele-specific expression. Baseline allele specificity of transcription in the maternal (MAT) or paternal (PAT) allele was not observed (NO) in the vehicle-treated sample (cutoff 80%), indicating erased imprinting in PGCs. Of those with baseline erasure, the expression or DNA methylation became more biased toward one parental allele in a few instances, indicating a lack of proper erasure of imprinted expression (see the last column).

### Effect of EDs on allele-specific DMR methylation in embryos derived from exposed prospermatogonia

*In utero* ED exposure can perturb the imprinting process in fetal germ cells at the time when genomic imprints are established at PAT DMRs and when MAT DMRs are protected from *de novo* DNA methylation. Such perturbation in the normal imprint establishment process in male germ cells would result in reduced *de novo* methylation at PAT DMRs in the prospermatogonia of G1 fetuses and reduced methylation in the paternally inherited allele in the soma of the G2 generation. Changes at MAT DMRs would mean lack of protection from DNA methylation in G1 prospermatogonia and consequently their increased methylation in the paternally inherited chromosome in the G2 generation. We anticipated that inherited epigenetic changes would result in aberrant DNA methylation of the same DMR in more than one organ.

**Figure 3:**
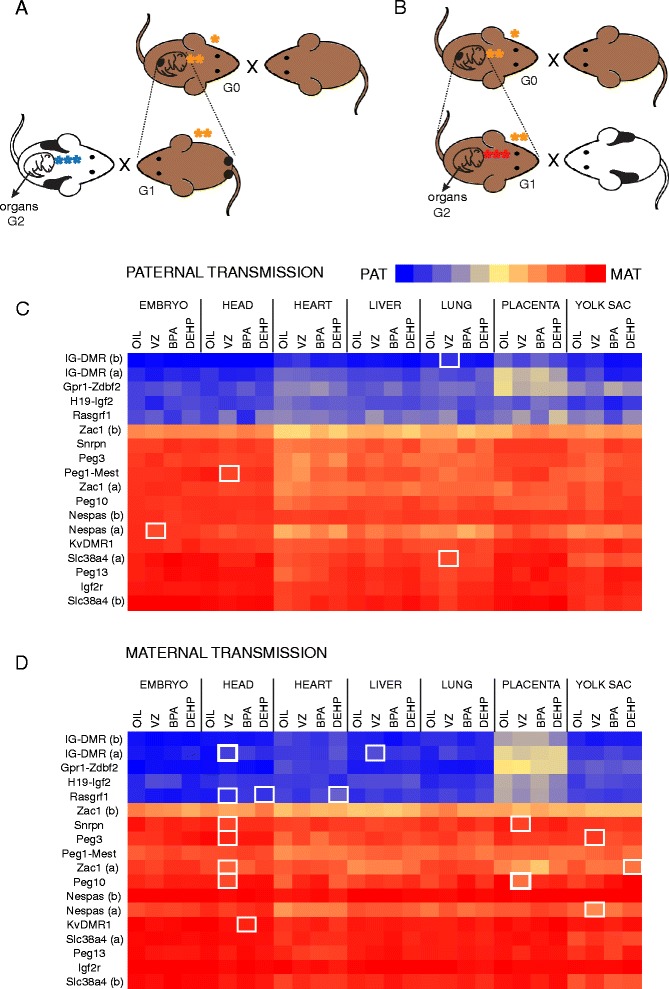
**Testing the effect of EDs on imprint establishment by assaying the allele-specific DNA methylation at DMRs in G2 embryos. (A)** *In utero* exposed male 129S1 G1 fetuses were grown to adulthood and mated with unexposed JF1 females; the resulting G2 fetuses (three blue stars) derived from exposed prospermatogonia. **(B)** *In utero* exposed G1 females were mated with unexposed JF1 males to generate G2 offspring (three red stars) which derived from exposed primary oocytes. G2 fetuses were dissected at 13.5 dpc to collect organs. **(C, D)** MIRA-SNuPE results of 13.5 dpc G2 embryo DNA samples after potential paternal and maternal transmission of aberrant DNA methylation at DMRs. The heatmap shows the percentage of average parental allele-specific methylation in organs; the color scale is as in Figure 2C. Letters in parentheses indicate different SNPs. Statistically significant differences relative to control (oil) greater than 5% and 10% are indicated by thin or bold rectangles, respectively (Student’s *t*-test, *P* <0.05).

To test whether ED exposure perturbs the imprinting process in the prospermatogonia, we first tested the allele-specific DNA methylation pattern at DMRs in the soma of the derived offspring. G1 129S1 male offspring were exposed *in utero* at the time of paternal imprint establishment (exposure B in Figure 1) on five consecutive days, 12.5 to 16.5 dpc, by oral gavage to pregnant G0 dams with one of the EDs or vehicle control (Figure 3A). When 129S1 exposed G1 males reached adulthood, they were mated with unexposed JF1 females to generate G2 offspring derived from the exposed prospermatogonia. The JF1 × 129 G2 fetuses were dissected at 13.5 dpc to collect head, heart, liver, lung, placenta, yolk sac, and embryo carcass. We isolated DNA and collected the CpG-methylated fraction using MIRA [40]. We quantified the percentage of parental alleles in the total methylated fraction at 14 DMRs (18 SNPs) using multiplex SNuPE assays [41]. The average percentage of paternal or maternal allele methylation is displayed in Figure 3C and Additional file 1. From 378 different experimental conditions (7 organs, 3 EDs, 18 SNPs), we found only four statistically significant differences (*P* <0.05) that were greater than 5% (Table 2), and no difference was higher than 10%. The CpG methylation was less paternally biased in G2 embryos at 13.5 dpc at the IG-DMR in the lung; the *Peg1-Mest*, *Nespas*, and *Slc38a4* DMRs were less maternally biased in the head, embryo carcass, and lung, respectively, after VZ treatment of the prospematogonia of G1 fetuses. Applying multiple testing, none of these changes were significant using the Bonferroni corrected *P* value (*P* <0.0027). Additional SNPs in two of these DMRs (IG-DMR and *Nespas*) did not substantiate the epigenetic aberration. We did not detect any change in the G2 soma after BPA or DEHP treatment of G1 prospermatogonia.

**Table 2:** **Significant changes between control and ED-treated samples after disturbing G1R DMR establishment**

							**% MAT allele**	
	**Organ**	**ED**	**Difference (%)**	**SD (%)**	**Student’s** ***t*** **-test** ***P*** **value**	**Imprinted base line**	**Oil**	**Treatment**
**G2 soma trough paternal germ line - DMR DNA methylation (Figure** 3 **C)**								
*Peg1-Mest*	Head	VZ	-9.1	2.523	0.038	YES (MAT)	95	86
*Nespas*	Embryo	VZ	-6.9	3.150	0.022	YES (MAT)	91	84
*Slc38a4* (b)	Lung	VZ	-5.9	1.747	0.006	YES (MAT)	94	88
IG-DMR	Lung	VZ	5.1	1.513	0.010	YES (PAT)	5	10
**G2 soma through maternal germ line - DMR DNA methylation (Figure** 3 **D)**								
*Peg10*	Placenta	VZ	-23.4	3.247	0.000	YES (MAT)	100	77
*Peg10*	Head	VZ	-9.5	2.146	0.035	YES (MAT)	95	85
*Nespas* (a)	Yolk sac	VZ	-8.5	4.049	0.023	YES (MAT)	80	71
*Snrpn*	Placenta	VZ	-8.4	4.675	0.049	YES (MAT)	95	86
*Snrpn*	Head	VZ	-7.7	2.521	0.020	YES (MAT)	94	86
*Zac1* (a)	Yolk sac	DEHP	-7.3	2.129	0.037	YES (MAT)	83	76
*KvDMR1*	Head	BPA	-7.2	1.497	0.001	YES (MAT)	100	93
*Peg3*	Head	VZ	-6.4	2.483	0.034	YES (MAT)	99	92
*Zac1* (a)	Placenta	VZ	-5.9	3.248	0.036	NO	73	67
*Zac1* (b)	Placenta	VZ	-5.7	2.239	0.018	NO	66	61
*Peg3*	Yolk sac	VZ	-5.6	2.541	0.022	YES (MAT)	88	83
*Zac1* (a)	Head	VZ	-5.5	1.528	0.036	YES (MAT)	85	79
*Rasgrf1*	Head	VZ	5.4	1.343	0.003	YES (PAT)	6	12
*Rasgrf1*	Head	DEHP	5.5	2.038	0.011	YES (PAT)	6	12
*Rasgrf1*	Heart	DEHP	6.4	1.413	0.003	YES (PAT)	15	22
IG-DMR (a)	Liver	VZ	9.0	1.087	0.002	YES (PAT)	9	18
IG-DMR (a)	Head	VZ	11.2	3.210	0.048	YES (PAT)	4	15
**G2 soma through paternal germ line - RNA transcripts (Figure** 4 **)**								
*Nesp*	Lung	DEHP	-14.3	2.166	0.001	NO	45	31
*Nesp*	Heart	DEHP	-11.9	3.157	0.042	NO	45	33
*Slc22a3*	Head	VZ	-7.8	2.097	0.027	NO	53	46
*Ascl2*	Liver	VZ	-7.1	3.323	0.043	NO	77	70
*Cdkn1c*	Embryo	BPA	-6.7	2.865	0.036	YES (MAT)	99	92
*Slc22a3*	Liver	VZ	-6.0	2.261	0.011	NO	57	51
*Slc22a3*	Liver	BPA	-6.0	2.925	0.025	NO	57	51
*Cdkn1c*	Embryo	VZ	-5.8	1.367	0.021	YES (MAT)	99	93
*Cdkn1c*	Embryo	DEHP	-5.8	0.535	0.014	YES (MAT)	99	93
*Slc22a3*	Lung	BPA	-5.7	2.710	0.031	NO	64	58
*Usp29*	Lung	BPA	5.7	1.424	0.040	YES (PAT)	3	9
*Gnasxl*	Heart	VZ	6.3	1.940	0.027	NO	26	32
*Rasgrf1*	Heart	VZ	9.0	0.722	0.006	YES (PAT)	18	27
*Gnasxl*	Lung	VZ	15.3	3.644	0.002	NO	31	46
**G2 soma through maternal germ line - RNA transcripts (** **Additional file** 3 **)**								
*Nesp*	Placenta	DEHP	-12.7	4.922	0.034	YES (MAT)	80	67
*Slc22a3*	Yolk sac	DEHP	-11.0	0.620	0.030	YES (MAT)	97	86
*Ascl2*	Liver	VZ	-9.4	1.324	0.034	NO	71	61
*Gnasxl*	Yolk sac	DEHP	-8.6	3.204	0.035	NO	52	43
**G3 soma through paternal germ line (lung and heart) - RNA transcripts (Figure** 5 **B)**								
*Cobl*	Heart	BPA	-28.0	2.289	0.004	NO	79	51
*Cobl*	Heart	VZ	-27.9	0.640	0.001	NO	79	51
*Cobl*	Lung	VZ	-19.7	2.033	0.005	NO	70	51
*Cobl*	Lung	BPA	-19.4	2.729	0.018	NO	70	51
*Phlda2*	Lung	VZ	-11.8	3.974	0.032	NO	79	68
*Cobl*	Heart	DEHP	-11.1	1.206	0.003	NO	79	68
*Rtl1/Rtl1as*	Lung	BPA	-10.0	1.649	0.021	YES (MAT)	90	80
*Tnfrsf22*	Heart	VZ	-9.9	1.942	0.026	NO	58	48
*Rtl1/Rtl1as*	Lung	VZ	-8.7	4.378	0.007	YES (MAT)	90	81
*Tnfrsf22*	Heart	BPA	-8.1	1.070	0.022	NO	58	50
*Tnfrsf22*	Lung	BPA	-7.6	1.305	0.023	NO	57	49
*Ascl2*	Heart	DEHP	-6.2	2.372	0.011	NO	54	48
*Tnfrsf22*	Lung	VZ	-5.5	2.043	0.025	NO	57	51
*Atp10a*	Heart	VZ	5.1	1.285	0.010	NO	48	53
*Sfmbt2*	Lung	VZ	6.5	0.690	0.003	NO	41	48
*Zim1*	Lung	BPA	6.7	1.473	0.010	YES (MAT)	90	97
*Snrpn*	Lung	VZ	9.9	2.488	0.019	YES (PAT)	11	21
*Slc22a3*	Lung	BPA	10.0	7.898	0.017	NO	50	60
*Nespas* (a)	Lung	VZ	10.3	2.375	0.036	YES (PAT)	3	13
*Ascl2*	Lung	BPA	12.7	3.520	0.000	NO	46	59
*Asb4*	Heart	VZ	27.2	8.101	0.019	NO	61	88
*Asb4*	Heart	BPA	27.2	2.591	0.015	NO	61	88

Note: (a) and (b) denote different SNPs for the given DMR or transcript.

Allele-specific DNA methylation or transcription was compared in 13.5 dpc embryos between ED- and vehicle-treated samples for each of the DMRs or imprinted transcripts, respectively. Changes in the average (n = 3) allele-specific features that were greater than 5% and were statistically significant (*P* <0.05) were tabulated and ordered according to the change in the maternal allele. Changes greater than 10% were rare. Baseline allele specificity of transcription or DNA methylation was observed (YES) or was not observed (NO) in the maternal (MAT) or paternal (PAT) allele in the vehicle-treated sample (cutoff 80%). Of those with baseline allele specificity, the expression or DNA methylation became less biased (relaxed) in a few instances (see the last column).

We also carried out the control experiment to test whether *in utero* EDs perturb the imprinting process in female fetal germ cells, which would result in increased *de novo* methylation at PAT DMRs in primary oocytes of G1 fetuses. That methylation could be carried into the soma of G2 generation in the maternally inherited allele of PAT DMRs. Perturbation at MAT DMRs would cause earlier DNA methylation in oocytes but would not be expected to change the final DNA methylation levels of the oocyte nor to increase DNA methylation in the maternally inherited chromosome in the G2 generation. We exposed the 129S1 G1 female offspring to an ED or vehicle control (Figure 3B) *in utero*. We crossed G1 adult females with unexposed JF1 males to generate G2 offspring, deriving from the exposed primary oocytes. We dissected the G2 fetuses at 13.5 dpc and collected seven organs/body parts for DNA isolation. We measured the parental allele-specific DNA methylation at 14 DMRs using MIRA-SNuPE assays (Figure 3D and Additional file 1). We found five significant changes in the maternal allele-specific methylation (>5%, *P* <0.05) at PAT DMRs (Table 2). The IG-DMR in the liver and head was affected by VZ exposure; the *Rasgrf1* DMR was affected in the head and heart by DEHP and in the head by VZ exposure of fetal oocytes. One of these, the IG-DMR in G2 liver was significant after Bonferroni correction of *P* value to *P* <0.0027. However, this aberration was not substantiated by another SNP in the same DMR.

We found 10 cases in which maternal allele-specific methylation decreased at MAT DMRs by at least 5% (*P* <0.05), and two remained significant after Bonferroni adjustment (*P* <0.0027). However, these aberrations in G2 soma cannot be explained by disturbed DNA methylation in fetal oocytes. Another layer of epigenetic regulation may have been disturbed to prevent the full *de novo* DNA methylation of MAT DMRs in growing oocytes after the birth of the G1 females. It is quite possible that ED exposure in G0 affects the ability of G1 females to reproduce by altering the uterine environment, and thus it may affect G2 independent of the germline. Since this experimental model is not as ‘clean’ when addressing germline inheritance as the male inheritance model (where there is no confounding uterine environment effect), these data should be interpreted with caution.

### Effect of EDs on allele-specific expression of imprinted genes in embryos derived from exposed prospermatogonia

**Figure 4:**
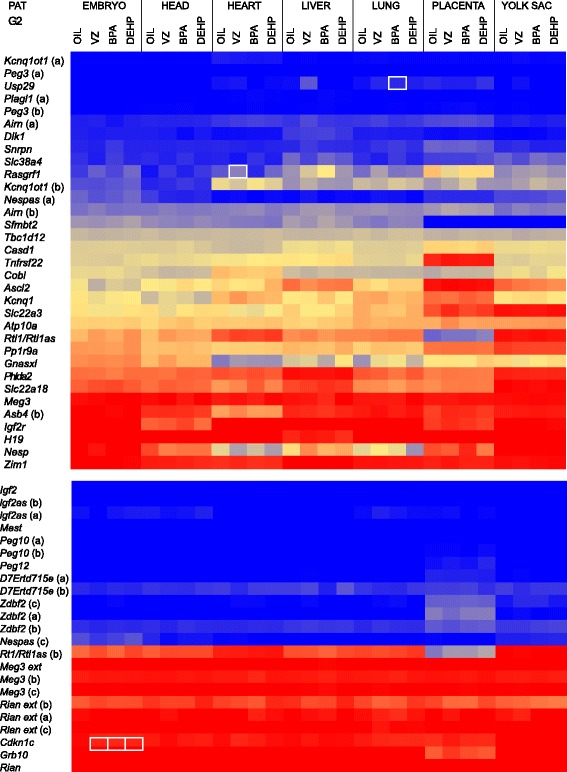
**Effect of EDs on allele-specific expression of imprinted genes in fetuses derived from exposed prospermatogonia.** Experimental design was as in Figure 3A. Results of RNA Sequenom allelotyping experiments of imprinted transcripts) are shown using the color scale as in Figure 2C; ‘(a)’ and ‘(b)’ denote independent SNPs in the same transcript. Average (n = 3) parental allele-specific transcription is displayed. Note the overall undisturbed allele-specific transcription. Statistically significant (*P* <0.05) differences between ED and control greater than 5% are indicated by thin rectangles. More groups of fetuses are shown in Additional file 2.

We expected that any epigenetic aberration - DNA methylation or other - occurring in G1 prospermatogonia at PAT or MAT DMRs would result in misexpression of imprinted genes in the soma of the G2 generation. To test for this possibility, we followed the experimental design shown in Figure 3A and measured parental allele-specific transcription in G2 embryo organs by RNA-SNuPE using multiplex Sequenom allelotyping assays at 56 SNPs. The results are provided in Additional file 1 and are displayed in two heatmaps (Figure 4). Analyzing three G2 individuals from each treatment, seven organs, and 56 SNPs, we generated 4,704 data points and compared the average values, finding five differences greater than 5% relative to control (Student’s *t*-test, *P* <0.05). None of these differences remained significant after multiple testing using Bonferroni correction of *P* values (*P* <0.0009). To test for possible inter-litter variations, we repeated this experiment with a larger number of G2 fetuses, selected organs, and selected SNPs, generating 1,350 data points (Additional files 1 and 2A); there were no differences greater than 5% relative to control (*P* <0.05). We conclude that allele-specific transcription was undisturbed in the G2 embryos derived from ED-exposed prospermatogonia.

We found no causative relationship between aberrations in allele-specific DNA methylation at DMRs and allele-specific transcription. For example, the IG-DMR was affected by VZ in lung (Figure 3C), but transcription of *Meg3*, *Rtl1/Rtl1as*, *Rian*, and *Rian extension*, all regulated by IG-DMR, were not altered there (Figure 4). Similarly, the *Nespas* DMR was altered by VZ in the embryo carcass, but the allele-specific transcription of *Nesp*, *Nespas*, and *Gnasxl* showed no change.

### Testing for transgenerational inheritance of aberrant imprinting

**Figure 5:**
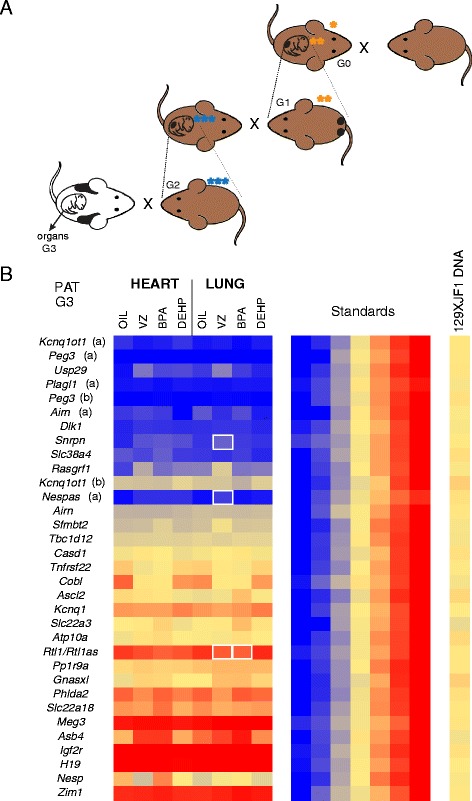
**Testing for transgenerational epigenetic inheritance of the aberrant imprinted expression. (A)** Breeding design to test whether ED-perturbed parental allele-specific transcription is transgenerationally inherited through the paternal germline to an unexposed generation. G1 male fetuses were exposed *in utero* to EDs or vehicle control (‘oil’) daily from 12.5 dpc to 16.5 dpc. After reaching adulthood, 129S1 G1 males were mated with 129S1 unexposed females to generate G2 offspring (3 blue stars), which derived from exposed prospermatogonia. At adulthood, G2 males were mated with unexposed JF1 females to generate G3 offspring, which were never directly exposed to EDs. JF1 × 129 G3 fetuses were dissected at 13.5 dpc to collect organs for RNA isolation. Parental-specific transcription was quantified in the total RNA using multiplex SNuPE assays. **(B)** Results of Sequenom allelotyping experiments using heart and lung tissue of the G3 generation; color scale as in Figure 2; letters in parentheses denote independent SNPs. Notice the lack of inherited changes from the exposed generation. More groups of fetuses are shown in Additional file 2. This Figure includes standards that are routinely included in the Sequenom runs (see Methods).

There were a few statistically significant perturbations in the transcription of imprinted genes in G2 heart and lung greater than 5% (Figure 4). We asked whether these could be passed from G2 individuals into the unexposed G3 generation. Our experimental design is shown in Figure 5A. We treated G1 male offspring *in utero* daily from 12.5 dpc to 16.5 dpc, when paternal imprint is occurring in prospermatogonia, with an ED or with vehicle control (‘oil’). We crossed adult G1 129S1 males with unexposed 129S1 females and allowed G2 males to reach adulthood. Then we crossed them with unexposed JF1 females to generate G3 offspring. Whereas G2 fetuses developed from ED-exposed germ cells, G3 fetuses developed from unexposed gametes. We dissected the heart and lung of JF1 × 129 G2 fetuses at 13.5 dpc and we quantified parental-specific transcription in the total RNA using multiplex SNuPE assays (Figure 5B and Additional file 1).

Changes that occurred in the G2 generation did not carry into G3. We found no statistically significant change (*P* < 0.05) greater than 5% in the paternal allele-specific transcription of *Usp29* in the heart (BPA) or *Rasgrf1* in the lung (VZ). We found three statistically significant changes (in *Snrpn*, *Nespas*, and *Rtl1/Rtl1as*) in G3 lung after VZ exposure of G1 prospermatogonia (Table 2), but these must be independent of the initial VZ exposure, because we did not find any misexpression of their transcripts in the G2 embryo. To test for inter-litter variation, we repeated this experiment using a larger number of G3 fetus livers and selected SNPs (Additional files 1 and 2B). In this dataset, we did not detect any difference greater than 5% as relative to oil control (*P* <0.05). Thus, our results do not support the hypothesis that ED-triggered epigenetic aberrations in imprint establishment of prospermatogonia are transgenerationally inherited.

### Effect of EDs on allele-specific expression of imprinted genes in embryos derived from exposed primary oocytes

We used the experimental design shown in Figure 3B to expose G1 129S1 female offspring to an ED or vehicle control *in utero*. We crossed G1 adult females with unexposed JF1 males to generate G2 offspring that were from exposed primary oocytes. We dissected JF1 × 129 G2 fetuses at 13.5 dpc, collected seven organs/body parts for RNA isolation, and measured the parental allele-specific DNA methylation at 33 SNPs using RNA-SNuPE assays (Additional files 1 and 3). We found four significant decreases in maternal allele-specific transcription of more than 5% (Table 2): *Slc22a3* and *Gnasxl* in the yolk sac after DEHP exposure, *Nesp* in the placenta (DEHP), *Ascl2* in the liver (VZ). None of these remained statistically significant after multiple testing using the Bonferroni corrected *P* value (*P* <0.0015). We found no causative relationship between aberrant allele-specific DNA methylation at DMRs and aberrant allele-specific transcription (Figure 3D and Additional file 3).

### DNA methylation establishment is undisturbed by EDs in prospermatogonia at DMRs

To test ED effects directly, we used the methylated CpG island recovery assay (MIRA) chip. We showed previously that MIRA-chip is sensitive to detect allele-specific DNA methylation at DMRs in mouse embryo fibroblasts (MEFs) [42] and to reveal dynamic DNA methylation changes at DMRs during fetal male germ cell development [19].

**Figure 6:**
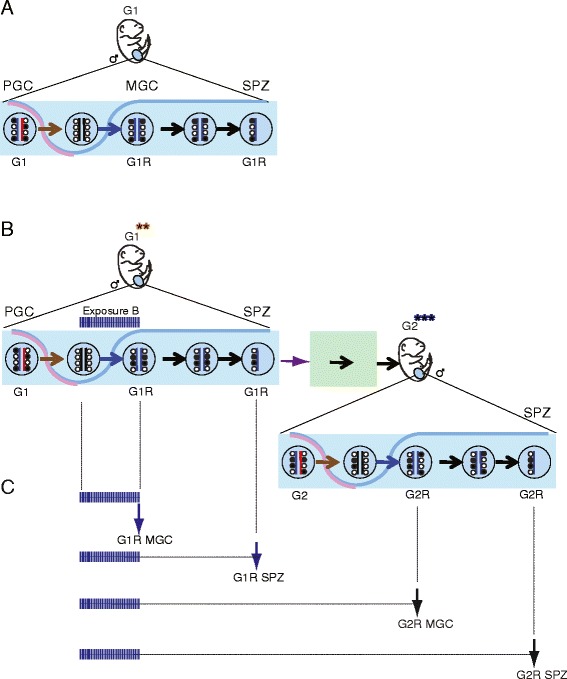
**Assessing** ***if in utero*** **exposure to ED results in transgenerationally inherited epigenetic aberration via the germline. (A)** Normal epigenetic remodeling the male germline. MGC undergo normal erasure and normal re-establishment of DNA methylation, producing reprogrammed G1 (G1R) MGCs and G1R spermatozoa. **(B)** Hypothetical situation where a G1 embryo (two orange stars) exposed to ED during the *de novo* DNA methylation process results in aberrant reprogramming of G1R MGCs. The aberrant DNA methylation pattern may be maintained in G1R spermatozoa. Aberrant DNA methylation pattern of G1R sperm may harm G2 embryos (three blue stars), by germline epigenetic inheritance. The right panel shows a hypothetical situation in which in the absence of further ED exposure, an aberrant DNA pattern is inherited from the G1R spermatozoa. This aberration fails to be erased in G2 PGCs, and is carried further into G2R prospermatogonia and G2R spermatozoa, which have not been exposed directly or indirectly; thus, persistence of the aberration in these cells would constitute transgenerational epigenetic inheritance. Note that the DNA methylation patterns are simplified, for example, they do not take into account remodeling during the zygote-early embryo stages (green box). **(C)** Timing scheme of the genome-wide mapping studies. G1 fetuses were exposed *in utero* during the establishment phase (Exposure B), and G1R and G2R fetal MGCs and adult spermatozoa were collected for analysis.

To detect immediate changes in DNA methylation in male germ cells at the time of exposure and when the changes are passed to the next generation, we mapped the methylation patterns from the *in utero*-exposed prospermatogonia and the emerging adult spermatozoa (Figure 6). After crossing a FVB dam and an OG2 father, we treated G1 male fetuses *in utero* from 12.5 dpc to 16.5 dpc to daily exposure to an ED or to vehicle control at the time of paternal imprint establishment in the prospermatogonia (Additional file 4A). We collected the exposed prospermatogonia at 17.5 dpc for DNA methylation analysis by FACS sorting. We allowed some G1 males to reach adulthood and collected their sperm (Additional file 4A). We isolated genomic DNA from the prospermatogonia and sperm and then we enriched for the methylated fraction using MIRA. We labeled the methylated fraction and hybridized it to custom Nimblegen microarrays that encompassed all known imprinted domains, as we did earlier [19].

**Figure 7:**
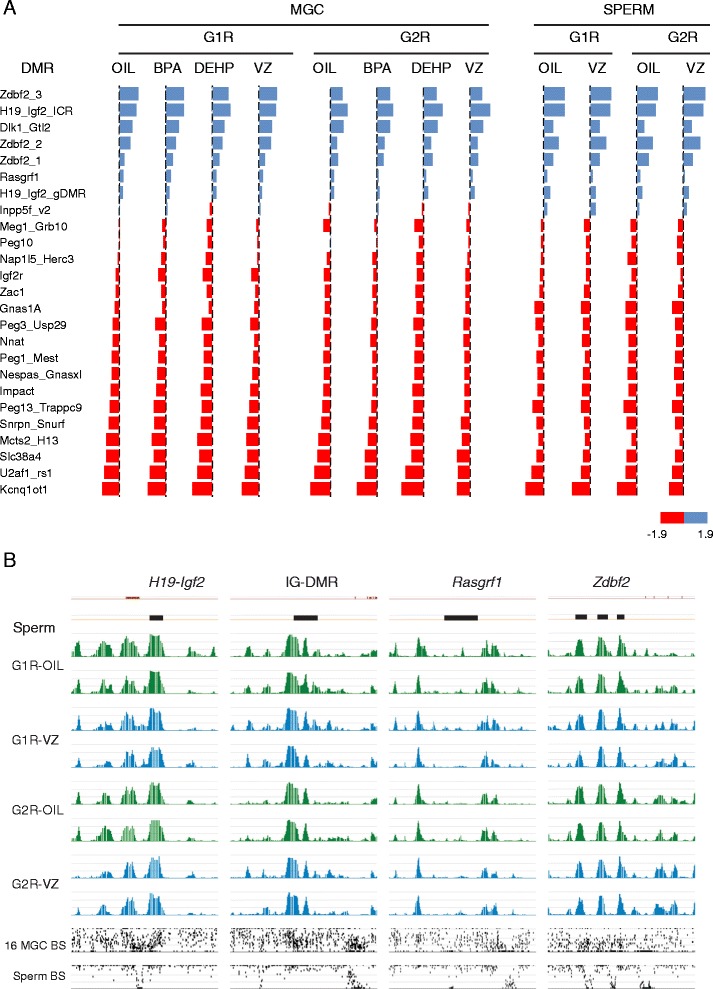
**DNA methylation establishment is undisturbed by EDs in prospermatogonia at paternally methylated imprinted DMRs.** DNA methylation was mapped in purified G1R and G2R prospermatogonia and spermatozoa using MIRA-chip and custom Nimblegen imprinting arrays. The mouse matings were conducted as depicted in Additional file 4. **(A)** Summary of MIRA-chip results at imprinted DMRs in custom imprinting arrays (groups 5 to 8). The average MIRA/input log2 ratios (n = 3 for MGCs and n = 2 for sperm) were calculated for known imprinted DMRs and are depicted with red (maternal and blue (paternal) flags in the range of -1.9 to +1.9. The full calculations are provided in Additional file 6. Note that paternally methylated DMRs have positive MIRA/input log2 ratios and maternally (MAT) methylated DMRs have negative MIRA/input log2 ratios, as expected. No MAT DMR exhibited increased methylation and no PAT DMR had decreased DNA methylation, using the cutoff values of ±5% and *P* <0.05 (Student’s *t*-test). **(B)** The MIRA profile is depicted at paternally methylated imprinted DMRs (black rectangles) in biological duplicate samples for VZ treatment or control. The DNA methylation signals of MIRA versus input DNA were plotted as -log10 *P* values in the range of 0 to 8.4. The average % DNA methylation levels at each CpG as determined by whole genome bisulfite sequencing (WGBS) are shown compared to that of normal MGCs at 16.5 dpc [18] and normal sperm [20]. Note, that DNA methylation at paternal DMRs is undisturbed by ED treatment in the exposed prospermatogonia and in the prospermatogonia of the next generation.

The methylation levels of PAT- and MAT-imprinted DMRs in prospermatogonia after ED treatment are shown in Figure 7A. In the controls (‘oil’), DNA methylation reprogramming occurred normally: G1-specific DNA methylation was erased and the reprogrammed (G1R)-type DNA methylation establishment was largely complete (Additional file 5) [19]. DNA methylation was similarly normal at imprinted DMRs in G1R sperm that developed from VZ-exposed G1R prospermatogonia (Figure 7B) and in ED-exposed G1R prospermatogonia (Figure 7A and Additional file 6). Strong peaks were observed at PAT DMRs (Additional file 5A) and valleys were found at MAT DMRs (Additional file 5B); none of these DMRs showed any change after ED exposure.

We also tested the G2R male germ cells of the next unexposed generation derived from exposed G1R propermatogonia (Additional file 4C and D). We exposed G1 male fetuses *in utero* daily from 12.5 dpc to 16.5 dpc to an ED or vehicle control. We allowed some G1 (FVBXFVB) males to reach adulthood and crossed them with OG2 females to obtain G2 fetuses (Additional file 4C). These fetuses derived from exposed G1R prospermatogonia, but their prospermatogonia that carried the G2R-type DNA methylation pattern had never been exposed to EDs. We collected the G2R-type prospermatogonia for DNA methylation analysis, and we allowed some VZ-exposed G2 (FVBXOG2) males to reach adulthood and collected their G2R-type spermatozoa (Additional file 4D). We analyzed the methylation patterns of these male germ cells at imprinted DMRs using MIRA-chip. We calculated the average MIRA/input log2 ratios (n = 3 for MGC and n = 2 for sperm) along known imprinted DMRs in the custom imprinting arrays (Figure 7A).

As expected, the paternally methylated DMRs had positive MIRA/input log2 ratios and the maternally methylated imprinted DMRs had negative ratios. We calculated the change in MIRA intensities between ED-exposed and control G1R prospermatogonia: no MAT DMR showed increased methylation and no PAT DMR showed decreased methylation, using cutoff values of ±5% and *P* <0.05. We found no changes in G2R-type prospermatogonia at PAT DMRs after any ED treatment (Additional file 6). There was no detectable change in G2R-type spermatozoa after VZ treatment (Figure 7B), and MAT DMRs were similarly unaffected (Additional file 6). Our data show no evidence for aberrant imprint establishment in propermatogonia after treatment with EDs, for its inheritance through the sperm, or for further transgenerational inheritance to unexposed offspring.

### Search for genome-wide changes in DNA methylation after ED exposure of G1 prospermatogonia

To find genome-wide immediate and persistent changes in DNA methylation, we used a genome-wide methylated CpG island recovery assay (MIRA) with Nimblegen CpG-island + promoter arrays (sample groups 1 to 4) and a custom, imprinting array (sample groups 5 to 7, as summarized in Additional file 7). The custom array included known imprinted genes, DMRs, control genes, IAP-flanking regions, and the Y chromosome. The log2 ratios of the MIRA/input signal from each hybridization value were quantified, the average values were calculated for the biological replicates, and these values were used in a multi-level data analysis (Table 3 and Additional file 8).

**Table 3:** **Analysis results for immediate and persistent changes in DNA methylation after ED exposure of G1 prospermatogonia**

			**Analysis level 1**		**Level 2**		**Level 3**		**Level 4**	
	**Sample A**	**Sample B**	**Sample A to B**	**Hits**	**Common in G1R and G2R**	**Hits**	**Common in MGCs and sperm**	**Hits**	**TGI**	**Hits**
Group 1: CpG_G1R_MGC	Oil	BPA	Test 1	112	Test A (test 1 v 7)	1 (1)				
	BPA	Oil	Test 2	72	Test B (2 v 8)	3 (3)				
	Oil	VZ	Test 3	129	Test C (3 v 9)	0 (0)	Test Q (3 v 13)	1 (1)	Test C v G	0 (0)
	VZ	Oil	Test 4	81	Test D (4 v 10)	0 (0)	Test R (4 v 14)	2 (1)	Test D v H	0 (0)
	Oil	DEHP	Test 5	130	Test E (5 v 11)	4 (1)				
	DEHP	Oil	Test 6	120	Test F (6 v 12)	6 (3)				
Group 2: CpG_G2R_MGC	Oil	BPA	Test 7	61						
	BPA	Oil	Test 8	98						
	Oil	VZ	Test 9	59			Test S (9 v 15)	4 (2)		
	VZ	Oil	Test 10	135			Test T (10 v 16)	2 (1)		
	Oil	DEHP	Test 11	89						
	DEHP	Oil	Test 12	167						
Group 3: CpG_G1R_SPERM	Oil	VZ	Test 13	76	Test G (13 v 15)	4 (4)				
	VZ	Oil	Test 14	76	Test H (14 v 16)	4 (3)				
Group 4:										
CpG_G2R_SPERM	Oil	VZ	Test 15	196						
	VZ	Oil	Test 16	99						
Group 5: Imp_G1R_MGC	Oil	BPA	Test 17	46	Test I (17 v 23)	0 (0)				
	BPA	Oil	Test 18	35	Test J (18 v 24)	0 (0)				
	Oil	VZ	Test 19	93	Test K (19 v 25)	1 (1)	Test U (19 v 29)	1 (1)	Test K v O	0 (0)
	VZ	Oil	Test 20	51	Test L (20 v 26)	1 (1)	Test V (20 v 30)	0 (0)	Test L v P	0 (0)
	Oil	DEHP	Test 21	83	Test M (21 v 27)	0 (0)				
	DEHP	Oil	Test 22	59	Test N (22 v 28)	1 (0)				
Group 6: Impr_G2R_MGC	Oil	BPA	Test 23	30						
	BPA	Oil	Test 24	45						
	Oil	VZ	Test 25	29			Test X (25 v 31)	0 (0)		
	VZ	Oil	Test 26	120			Test Y (26 v 32)	4 (4)		
	Oil	DEHP	Test 27	27						
	DEHP	Oil	Test 28	111						
Group 7: Impr_G1R_SPERM	Oil	VZ	Test 29	56	Test O (29 v 31)	3 (2)				
	VZ	Oil	Test 30	58	Test P (30 v 32)	1 (0)				
Group 8: Impr_G2R_SPERM	Oil	VZ	Test 31	114						
	VZ	Oil	Test 32	93						

After *in utero* exposure of G1 MGC to vehicle control (‘oil’) or one of the EDs, the level of DNA methylation was measured in reprogrammed G1R and G2R fetal MGCs and adult spermatozoa by MIRA-chip and Nimblegen microarrays. CpG-promoter arrays were used in groups 1 to 4 and custom imprinting arrays were used in groups 5 to 7. At the first level of analysis, we performed 32 tests comparing the log2 ratio MIRA versus input values. To detect a change between sample A and B, peaks were identified in the average value (n = 3 for MGC and n = 2 for sperm) of A samples first and were compared with the MIRA intensity of the average of sample B at the same locations. The number of hits where a change occurred with greater than ±5% and Fisher’s exact test *P* value (*P* <0.05) were tabulated. At level two, we compared the results of two level-1 tests (in parentheses) to find common changes between G1R and G2R in MGCs (Tests A-F and I-N) and in sperm (Tests G-H and O-P); the number of common hits is provided and those that changed in the same direction are given in parentheses. At level three, we compared the results of two level-1 tests to find common changes between MGC and sperm in the same generation, G1R (Test Q-R and U-V) or G2R (S-T and X-Y). At level four, we compared the results of four level-1 tests in search for true transgenerational epigenetic inheritance: immediate effect in G1R MGCs, maintained into G1R sperm, G2R prospermatogonia, and G2R sperm, thereby having the potential to affect G3 soma. Note the low number of hits in levels 1 to 3 and lack of hits at level 4, even at the low cutoff values applied.

At the first level we tested for changes between pairs of experimental and control samples. We compared the log2 ratio MIRA versus input values for each sample ‘A’ with the corresponding sample ‘B’. We identified peaks in sample ‘A’ first and calculated the difference between ‘A’ and ‘B’ in samples at these locations. We performed this analysis two ways, calling the experimental sample ‘A’ and control sample ‘B’ and vice versa, to ensure that a change would be detected at each peak even when it occurred only in ‘A’ or in ‘B’. We chose very low cutoff values - changes greater than ±5% with Fisher’s exact test *P* value (*P* <0.05) - to allow the higher-level analyses of a larger set of primary hits. At the second analysis level, we compared the results of two level-1 tests to find common changes between G1R and G2R samples. At level 3, we compared the results of two level-1 tests to find common changes between MGCs and sperm in the same generation. At level 4, we compared the results of four level-1 tests: an immediate effect in G1R MGCs that persisted into G1R sperm, G2R prospermatogonia, and G2R sperm, thereby having the potential to affect the G3 soma.

**Figure 8:**
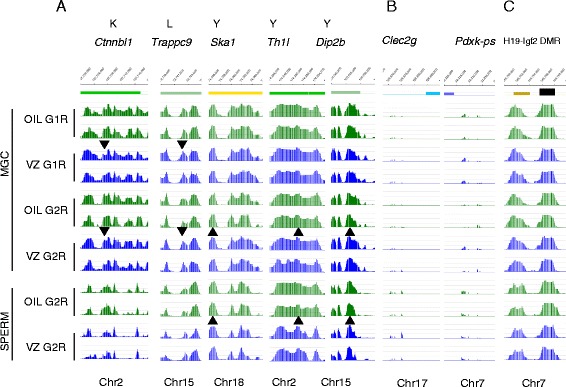
**Selected top hits of transgenerationally inherited DNA methylation aberrations.** Prospermatogonia of G1 fetuses were treated with ED or oil control *in utero* as depicted in Additional file 4. Next, DNA methylation was mapped using MIRA-chip and custom Nimblegen arrays in purified G1R and G2R prospermatogonia at 17.5 dpc in triplicate and in adult spermatozoa in duplicate. Immediate and persistent changes are tabulated in Table 3 and Additional file 8. **(A)** Selected top persistent hits are shown from the analysis in duplicates labeled at the top according to the comparisons in Table 3 and marked with arrowheads (up for increase and down for decrease) **(B)** A selected IAP-flank region where common changes were detected in MGC samples between G1R and G2R at and between G2R MGC and G2R sperm. **(C)** The *H19-Igf2* imprinted DMR is shown as a positive control for the DNA methylation signal (black rectangle). DNA methylation signals of MIRA versus input DNA are plotted as -log10 *P* values ranging from 0 to 8.3 for experimental and control replicate samples. Note that these top changes are minor and not highly significant.

We found very few hits at levels 2 to 3 and no hits at level 4, despite the low cutoff values (Table 3 and Additional file 8). Some examples of the best hits of the level 2 and 3 analyses are shown in Figure 8A; these hits are unimpressive and exist at regions with generally low DNA methylation.

We inspected the locations of the best hits in a recent study of G3R sperm after initial VZ exposure in G1 MGCs [43]. We found no change in G1R and G2R MGCs and sperm at *Mro* and found a complete lack of DNA methylation at *Elf3* (Additional file 9). One possible explanation for the discrepancies can be methodological differences. Whereas we hybridized each biological replicate against input and measured methylation levels along chromosomes, Guerrero-Bosagna hybridized control and experimental samples against each other, measuring differences in each chip, thus lacking information about DNA methylation levels at specific regions.

### Search for changes in DNA methylation at IAPs

Interesting targets for ED-caused aberrant DNA methylation may be intracisternal A-particle elements (IAPs), which retain substantial DNA methylation through the germline erasure process and thus can lead to transgenerational inheritance (TGI) of epigenetic changes [18,44]. Because most IAPs become highly methylated by the spermatozoa stage, it may be the rare unmethylated IAPs that carry specific information to the respective transcripts they may affect, and in turn, these may depend on the protection by H3K4 methylation [19]. We calculated the average DNA methylation along each unique 1-kb-long IAP-flanking region in the custom imprinting arrays and identified the DNA methylation changes at these regions between the exposed and control-treated samples with cutoff values of ±5% and *P* <0.05 (Additional file 10).

To reveal TGI of DNA methylation aberrations, we searched for common changes at IAP-flank regions between G1R and G2R in MGC samples after the initial exposure of G1R MGCs to VZ; those changes are shown with chromosomal coordinates in Additional file 11. Similarly, we identified the changes at IAP-flanking regions that were common between VZ-exposed G1R prospermatogonia and G1R sperm or between G1R and G2R sperm (Additional file 11 and Figure 8B). We also identified the common changes in G1R and G2R prospermatogonia that occurred after initial DEHP or BPA exposure in G1R (Additional file 10). The changes were small, often occurred in the opposite direction, and seldom mapped to gene promoters.

Our MIRA-chip results collectively suggest that BPA, DEHP, and VZ at the given doses have negligible immediate and persistent effects on the *de novo* DNA methylation process in mouse G1R prospermatogonia at CpG islands, promoters, imprinted DMRs, IAPs, and along the Y chromosome.

### Search for immediate and persistent changes in genome-wide transcription after ED exposure of G1 prospermatogonia

Although we found no evidence for TGI at the level of DNA methylation, other mechanisms such as histone modifications, histone variants, and long non-coding RNAs also participate in gene regulation and may transmit epigenetic aberrations between generations. Such aberrations are likely manifest in altered gene expression patterns. Therefore, we carried out Affymetrix microarray hybridization experiments using RNA from FACS-sorted 17.5-dpc fetal FGCs and MGCs exposed *in utero* to BPA, DEHP, VZ, or vehicle control. To find immediate direct responses to ED exposure, we analyzed G1R fetal oocytes and prospermatogonia. To find persistent changes, we analyzed G2R prospermatogonia. For the summary of samples, see Additional file 7.

**Figure 9:**
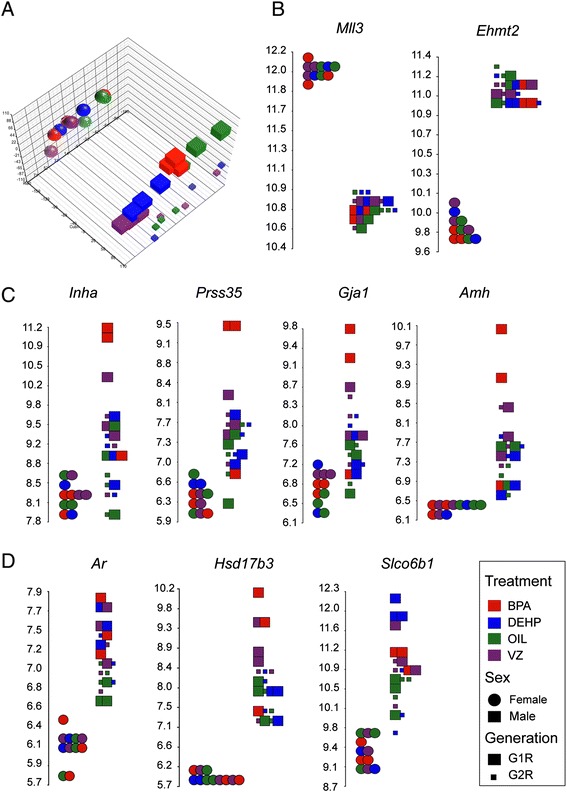
**Search for immediate and persistent changes in RNA levels after ED exposure of G1 prospermatogonia.** G1R prospermatogonia were purified at 17.5 dpc from female or male fetuses exposed *in utero* to BPA, DEHP, VZ, or vehicle control (‘oil’) and G2R prospermatogonia were also purified. Trancription of mRNA was measured using Affymetrix 1.0ST chips and the data were statistically analyzed in the Partek suite. **(A)** Principal component analysis of the samples. Note that the major principal component is sex and the second is generation. In addition, G1R male samples are separated by ED treatment. **(B)** Selected control transcripts are shown with known reciprocal expression patterns in the two sexes. **(C)** Selected top hits are shown that are upregulated in BPA-exposed G1R prospermatogonia. These genes are known targets of β-estradiol. **(D)** Selected top hits in VZ- and DEHP-exposed G1R propsermatogonia. Note that the androgen pathway is affected.

We found that sex was the main dividing parameter in principal component analysis (PCA) (Figure 9A), as expected based on our previous transcription profiling of fetal germ cells using RNA-seq [19]. We confirmed the male or female germ cell-specific transcription of epigenetic modifiers: *Mll3* and *Ehmt2* were highly expressed in FGCs and MGCs, respectively, and neither showed any change in transcription in response to any of the EDs (Figure 9B). G1R and G2R male germ cells were slightly separated from each other in the PCA (Figure 9A), likely because these samples were run at different times or because of the reciprocal genome composition (FVB × OG2 and OG2 × FVB, respectively) of these cells. Importantly, G1R prospermatogonia samples were clustered based on treatment type: BPA samples shifted slightly from control oil samples, and groups of DEHP and VZ samples shifted further away. Such clustering of samples was not apparent in G1R female germ cells or G2R male germ cells (Figure 9A). Using sex as an internal biological difference, we compared G1R female control oil samples with G1R male controls and found 11,848 statistically significant differences (6,552 up in female, 5,296 up in male) between sexes using 1.5-fold change and a false discovery rate (FDR) of 0.05 (Table 4). Similar numbers were found when comparing the respective female and male samples after BPA, DEHP, and VZ treatment. Notably, we found no overlap in the female up hits in the controls with the female down hits in any of the samples. These comparisons assured us that the data quality and analysis pipeline allows us to detect inherent biological differences in this sample set.

**Table 4:** **Search for immediate and persistent changes in RNA transcript levels after ED exposure of G1 prospermatogonia**

				**Cutoff 1.5-fold,** ***P*** **<0.05**			**Cutoff 1.05-fold,** ***P*** **<0.05**		
	**Condition 1**	**Condition 2**	**Probes** ^**a**^	**Probes**	**Unique**	**Common G1R-G2R**	**Probes**	**Unique**	**Common G1R-G2R**
Female-Male	G1R BPA F	G1R BPA M	12,310						
	G1R DEHP F	G1R DEHP M	11,886						
	G1R VZ F	G1R VZ M	11,877						
	G1R OIL F	G1R OIL M	11,848						
ED-OIL	G1R BPA F	G1R OIL F	0	230	14		1,592	933	
	G1R DEHP F	G1R OIL F	0	217	**50**		2,284	1,590	
	G1R VZ F	G1R OIL F	0	194	20		2,133	1,412	
	G1R BPA M	G1R OIL M	0	247	**125**		2,552	1,842	
	G1R DEHP M	G1R OIL M	**7**	**292**	**48**	1 (0/1)	**5,210**	**3,639**	325 (77/325)
	G1R VZ M	G1R OIL M	**2**	**549**	**61**	8 (0/8)	**6,499**	**4,295**	284 (30/284)
	G2R DEHP M	G2R OIL M	0	199	12	1 (0/1)	2,456	1,482	325 (77/325)
	G2R VZ M	G2R OIL M	0	186	16	8 (0/8)	1,912	1,044	284 (30/284)
Average number of probes				264	43		3,080	2,029	

^a^Cutoff: 1.5-fold, FDR *P* <0.05.

Prospermatogonia were purified at 17.5 dpc from female (F) or male (M) fetuses exposed *in utero* (G1R) to BPA, DEHP, VZ, or vehicle control (‘oil’). Prospermatogonia were also purified from the next generation (G2R). Trancription of mRNA was measured using Affymetrix 1.0ST chips. Transcription differences were detected between conditions 1 and 2. The hits are tabulated according the cutoff values, shown in the heading, as probes and unique transcripts. Bold numbers are higher than average among ED treatments for the given cutoff values. Common changes were detected between the unique transcripts that change in G1R and G2R samples for the same treatment; the numbers of those that changed in the same direction are in parentheses.

We generated the counts of differentially expressed probes and unique transcripts using Ingenuity Pathway Analysis (IPA) for each ED exposure versus control (Table 4). Using cutoff values of 1.5-fold change and FDR *P* <0.05, we found seven probes and two probes in the G1R DEHP male and G1R VZ male samples, respectively, but no probes in the other samples. When we relaxed the statistical cutoff value to simply *P* value <0.05 (with 1.5-fold difference), we found an average of 264 probes per condition; using cutoffs of 1.05-fold and *P* value = 0.05, we found an average of 3,080 probes per condition. We considered these relaxed cutoff values rather loose and likely to result in false positives. However, at any cutoff value, G1R DEHP versus control male and G1R VZ versus control male samples consistently yielded larger numbers of differences than the average numbers of other comparisons. In addition, the number of unique transcripts was higher than average in G1R DEHP versus control female and G1R BPA versus control male samples. This suggested that there are subtle transcription changes in these four conditions that are caused by ED exposure. IPA analysis revealed that the transcription changes affected the reproductive or endocrine system in each case. Interestingly, IPA identified β-estradiol as the upstream regulator for G1R BPA MGCs, with a probability of 4.57 × 10^-19^; four transcripts of the top 10 are regulated by β-estradiol (Figure 9C). Selected top hits in the male G1R VZ and DEHP samples are depicted in Figure 9D. Hydroxysteroid (17-beta) dehydrogenase 3 (*Hsd17b3*) was upregulated by BPA and VZ, while androgen receptor (*Ar*) was upregulated by VZ and DEHP. These transcripts are interesting because they play roles in the androgen pathways.

We noticed that none of the G1R top changes were detected in G2R (Figure 9). To further search for persistent changes in transcription, we compared the lists of differential expression in G1R MGCs with the respective G2R samples (Table 4). At the cutoff values of 1.5-fold and *P* <0.05, we found only one and eight common changes for DEHP and VZ, respectively, but none of these unique transcripts changed in the same direction. At the cutoff values of 1.05-fold and *P* <0.05, we found 325 and 284 common changes for DEHP and VZ, respectively. However, only 77/325 and 30/284 unique transcripts changed in the same direction, fewer than expected by chance (Additional file 12). When we considered all probes with common changes between G1R and G2R, Fisher’s exact tests revealed that a significantly greater number of the common changes occurred in the opposite direction. For DEHP, G0 downregulated (G1:Down) genes are significantly more likely to be in G1:Up (1.8×, *P* = 2.6 × 10^-5^), and G0:Up genes are significantly more likely to be in G1:Down (4.0×, *P* < 2.2 × 10^-16^). For VZ, G0:Down genes are significantly more likely to be in G1:Up (5.4×, *P* <2.2 × 10^-16^), and G0:Up genes are significantly more likely to be in G1: Down (7.2×, *P* <2.2 × 10^-16^).

In summary, ED exposures caused relatively small effects compared with those resulting from sex and generation number. The changes, however, affected hormonal pathways for the G1R BPA, G1R DEHP, and G1R VZ male samples, confirming that the drugs have reached their targets in fetal germ cells. The ED treatments had more effect in prospermatogonia than in fetal oocytes, likely because male germ cells undergo epigenetic establishment phase at the fetal stages and the establishment process may be vulnerable to environmental disturbances. VZ and DEHP caused more transcription differences in male germ cells than BPA, possibly because male germ cells may be more responsive to androgen signaling than to estrogen signaling. Notably, no treatment effect on transcription persisted from G1R to G2R, suggesting that the germ line is capable of rebounding from epigenetic effects caused by EDs.

## Discussion

To date no molecular evidence exists in mammals that fulfills the following criteria of TGI after *in utero* exposure: (1) an epigenetic aberration is detected in the exposed fetal germ cells; (2) the aberration is retained in the gamete; and (3) the same aberration is detected in the germ cells of the next generation. The aim of the present study was to systematically and rigorously evaluate the effects of EDs on global epigenetic reprogramming in the male mouse germ line after *in utero* exposure. We selected EDs that were reported to cause epigenetic aberrations, and focused on three EDs that affect estrogenic and androgenic pathways. Indeed, our exposures have reached the fetal germ cells, as we detected specific changes of transcription in G1R MGC that could be expected based on the known estrogenic properties of BPA and anti-androgenic properties of DEHP and VZ. We found that BPA caused the activation of estrogen-responsive genes, whereas VZ and DEHP induced elevated *Hsd17b3* and *Ar* transcripts, respectively, in the exposed G1R MGCs. However, these changes did not persist into the G2R MGCs. We investigated global DNA methylation changes at CpG islands, promoters, imprinted DMRs, and IAP repeats, and we did not find evidence for persistent changes between G1R and G2R prospermatogonia.

Even if we encountered a very persistent epigenetic aberration that occurred in the fetal germline of one sex and was maintained through several generations, we would expect to find a dilution of this effect with every generation, because meiosis results in haploid gametes and the chance of getting this allele is halved in every consecutive generation. The only exemption to this rule would involve the Y chromosome. We found no persistent changes in global transcription or DNA methylation between the exposed G1R and the next G2R generations along the Y chromosome.

TGI is perhaps easier to explain in organisms like *C. elegans*, where the germline is set aside at the zygote stage [45]. Even though the *C. elegans* germline also undergoes global epigenetic remodeling that mainly involves erasure and re-establishment of active histone modifications [46], any epigenetic aberration could be more easily inherited in the daughter cells that remain in the germ lineage. Indeed, in *C. elegans*, deficiencies in the H3K4me3 chromatin modifiers in the parental generation extended the life span of three generations in the wild type descendants [47]. However, in mammals, the germline develops from progenitors in the proximal epiblast and these cells have already differentiated away from the pluripotent state. Any epigenetic aberration has to resist two global waves of epigenetic reprogramming: the first occurs after fertilization in the zygote-preimplantation stage and the second in the primordial germ cells. Both waves involve erasure of the old patterns and the re-establishment of new patterns. These two global waves of remodeling must be the mechanism that removes epigenetic damage caused by the environment, ensuring that these are not inherited into the soma of the G3 generation. It is tempting to speculate whether avoiding TGI of environmental aberrations played an evolutionary role in the development of dual global reprogramming events in mammals.

## Conclusions

Our data show that whereas endocrine disruptors affect the transcription and DNA methylation state of exposed germ cells, these changes are not found in the germ cells of the subsequent generation. The genome-wide epigenetic remodeling processes in the next generation are robust, allowing the mammalian germline epigenome to recover from the effects of *in utero* exposure to endocrine-disrupting chemicals.

## Methods

### Ethics statement

Housing and care of the animals were consistent with Public Health Service Policy, the NIH *Guide for the Care and Use of Laboratory Animals*, and the Animal Welfare Act. All of the animal experiments were approved under protocol ID 91023 by the IACUC, City of Hope.

### Treatment regimens for the selected EDs

Mouse transgenic line TgOG2 [28] and inbred FVB, 129S1, and JF1 mice were used in the various studies. Animals were housed in polypropylene cages and received a special verified diet, 5 K96 (TestDiet), as recommended by the NIH for animal studies involving hormone-like chemicals. Drinking water was provided in glass bottles and was purified on a carbon filter (Filter Cartridge Hi-Cap Carbon 9-3/4 ID #: 2100-1970-102 from Edstrom Direct) just upstream of the bottle filler. Pregnant females in generation 0 (G0) were gavaged with EDs daily for 5 days starting at 8.5 dpc for the erasure study or at 12.5 dpc for the establishment study.

The EDs used were vinclozolin (ChemService Catalog no. PS-1049; Sigma, USA), bis(2-ethylhexyl) phthalate (Selectophore, (DEHP), Catalog no. 80030; Fluka/Sigma Inc.), and bisphenol A (Catalog no. 239658; Sigma Aldrich Inc.). All three EDs were dissolved/suspended in tocopherol-stripped corn oil vehicle (MPI Catalog no. 0290141584). Control animals were treated with the oil vehicle alone [48,49]. The oral doses for VZ (100 mg/kg/day), BPA (0.2 mg/kg/day), and DEHP (750 mg/kg/day) were the same as in our previous study [32]. These doses to pregnant mice are known to reach and to affect the fetus. VZ, for example, given by oral gavage at 10 or 50 mg/kg doses daily between 13.5 dpc and 17.5 dpc resulted in morphological changes in mouse fetuses at 19.5 dpc, including feminization of males (hypospadias) and virilization (longer urethras) of females together with altered gene expression in the genital tubercles [50,51]. Even a low dose of 1 mg/kg, VZ administered to pregnant mice in drinking water between 15 and 22 dpc resulted in decreased anogenital distance, prostate weight, relative testis weight, sperm count, and acrosomal state; caused increased sperm head abnormalities and pathology of the testes; and affected the expression of selected genes in male G1 offspring [52]. *In utero* and lactational exposure to DEHP at 750 mg/kg/day caused severe male reproductive system toxicity in rats including reduced sperm count and testicular malformations [48]. In mice, DEHP administration to pregnant mouse dams in food at 0.01% or 0.03% [53] (an estimated 0.75 to 1 g/kg or 2.25 to 3 g/kg/day dose, respectively), caused fetal defects and reduced the number of litters and number of live pups. At such daily doses, the DEHP metabolite MEHP clearly showed dose-dependent accumulation in 18.5-dpc fetal livers [54]. Oral administration of a single dose of 10 mg/kg BPA to pregnant mouse dams resulted in 0.01 mg/L BPA or 0.03 mg/L total BPA (including metabolite) in 15.5-dpc fetuses in the first hour [55], and three consecutive daily doses were additive. Feeding BPA (0.02 mg/kg) to pregnant mice at 11.5 to 17.5 dpc significantly decreased sperm production in the male offspring [8] and disturbed oocyte development and meiosis in female fetuses [56]. The same dose of BPA at 11.5 to 14.5 dpc lead to a large number of subtle changes of transcription in the fetal ovary [57].

### Purification of germ cells

Using a MoFlo or Aria III flow cytometer, germ cells (GFP-positive), and somatic cells (GFP-negative) were flow-sorted from embryonic or fetal gonads at 9.5, 13.5, or 17.5 dpc based on germ cell-specific EGFP expression in the TgOG2 transgenic mouse line [28] as described previously [58]. Phenol red was excluded from the M2 medium. Spermatozoa were collected from the cauda epididymis of adult FVB males and the motile fraction was used for MIRA-chip analysis.

### DNA isolation and methylated CpG island recovery assay (MIRA) and MIRA-chip

Genomic DNA was isolated from fetal germ cells by proteinase K digestion and phenol-chloroform extraction. Contaminating RNA was removed by RNAse treatment (Roche). RNA-free genomic DNA was sonicated to 300 to 800 bp using a standard Bioruptor water bath sonicator (Diagenode). Sonicated DNA (500 ng) was used for MIRA, as described previously [40]. The methylated fraction was captured using recombinant MBD2b and MBD3L1 proteins as described earlier [59] and was amplified by ligation-mediated PCR as previously described [42]. CpG-promoter arrays and custom-designed tiling arrays (110228_MM9_PS_ChIP), including all known imprinted domains and IAP flanking regions (Roche/NimbleGen), were used for the CpG methylation profile analysis [19]. Amplified MIRA DNA fractions were compared with amplified input DNA. Data were extracted from scanned images by using NimbleScan 2.3 extraction software (NimbleGen Systems).

### Detecting DNA methylation changes in MIRA-chip

To identify statistically significant changes caused by ED treatment, we compared treated versus oil control and also oil control versus treated samples in triplicates. To identify individual peaks in sample A, probes were considered positive if their normalized log2 ratio was above the 95th percentile of all probes on the array, and peaks were defined as four or more consecutive positive probes allowing one gap. The common peaks were identified between the triplicates of sample A and their mean value was compared to that of sample B. DNA methylation changes were identified based on the average log2 ratio signal difference between sample A and sample B, using cutoff values of ±5% (minimum 1.05-fold increase or 0.95-fold decrease) and Fisher’s exact *t*-test *P* value, *P* <0.05. To compare DNA methylation levels at DMRs and IAPs, we calculated the average log2 MIRA/input values along these sequences and compared them to detect changes between conditions with the cutoff values of ±5% and *P* <0.05 (Student’s *t*-test).

### RNA isolation and Affymetrix microarray hybridization

RNA was isolated from germ cells using TRIzol (Qiagen) extraction followed by ethanol precipitation. The RNA samples were processed in the City of Hope’s Microarray Facility using Mouse Gene 1.0 ST arrays (Affymetrix). Fold-change values were calculated based upon the least-squares mean using Partek Genomics Suite. Prior to statistical analysis, data were normalized using robust multichip average (RMA) normalization [60]. We used an ANOVA model with linear contrast to identify genes that have a change under condition A relative to condition B, with specific statistical significance and fold-change values, as specified in the text. We considered interactions between treatment, sex, and generation. Microarray data were deposited in GEO: Super series GSE59543.

### RNA isolation and reverse transcription-PCR

RNA was isolated from germ cells and various organs/body parts using RNA-Bee (Tel-Test). Contaminating DNA was removed with the DNA-free Kit (Ambion). cDNA was reverse-transcribed from total RNA using SuperScript III First-Strand Synthesis kit (Invitrogen).

### Analysis of allele-specific DNA methylation and gene expression by Sequenom allelotyping

Allele-specific DNA methylation and gene expression was measured by multiplex SNuPE assays [29,61] on the Sequenom platform, as we have done previously [32,41,62,63]. These assays are based on single nucleotide polymorphisms (SNPs) that distinguish between the inbred JF1/Ms (JF1) and 129S1 (129) mouse strains, or between the JF1 and the TgOG2 (OG2) transgenic mouse strains. Each SNuPE primer (UEP) abuts a SNP in a target DMR/transcript, and the incorporating nucleotides differ in molecular mass between the parental alleles. The abundance of the extended UEP is quantified by mass spectrometry. MIRA-enriched samples or amplified cDNA samples were spotted onto a 384 SpectroCHIP Array. Automated spectra acquisition was performed in a MassArray Compact mass spectrometer (Sequenom) using the Spectroacquire program (Sequenom) and was analyzed by MassArray Typer v3.4. RNA-mixing standards were routinely run to verify linear response in measured versus input allele-specific transcription: for example, total RNA from JF1 and 129 embryos was mixed in different percent ratios (0:100, 10:90, 30:70, 50:50, 70:30, 90:10, and 100:0) before cDNA preparation and Sequenom allelotyping. A true heterozygote DNA sample was used for DNA skew correction; a 50:50 RNA mix was used for RNA skew correction. The percentage of DNA methylation or transcription of each allele in the total methylation or expression was calculated at each given SNP. Primers are listed in Additional file 13.

### Statistical analysis

All statistical test *P* values refer to Student’s *t*-test unless otherwise noted.

## Additional files

Additional file 1:
**Sequenom data tables.** **(A)** Testing the effect of endocrine-disrupting chemicals (EDs) on imprint erasure. The full dataset depicted in Figure 2 is provided. **(B)** Testing the effect of EDs on the imprint establishment in prospermatogonia by measuring allele-specific DNA methylation at DMRs in G2 soma. The full dataset depicted in Figure 3C is provided. **(C)** Testing the effect of EDs in fetal primary oocytes by measuring allele-specific DNA methylation at DMRs in G2 soma. The full dataset depicted in Figure 3D is provided. **(D)** Effect of EDs on allele-specific expression of imprinted genes in G2 embryos derived from exposed prospermatogonia. The full dataset depicted in Figure 4 is provided. **(E)** Effect of EDs on allele-specific expression of imprinted genes in larger number of G2 embryos derived from exposed prospermatogonia. The full dataset depicted in Additional file 2, panel A is provided. **(F)** Testing for transgenerational epigenetic inheritance of aberrant imprinted expression in G3 soma. The full dataset depicted in Figure 5 is provided. **(G)** Testing for transgenerational epigenetic inheritance of aberrant imprinted expression in larger number of G3 fetuses. The full dataset depicted in Additional file 2, panel B is provided. **(H)** Effect of EDs on allele-specific expression of imprinted genes in embryos derived from exposed primary oocytes. The full dataset depicted in Additional file 3 is provided. Additional file 2:
**Analysis of larger number of fetuses.** **(A)** Effect of EDs on allele-specific expression of imprinted genes in fetuses derived from exposed prospermatogonia. Average parental allele-specific transcription in different body parts/organs of three 13.5 dpc G2 fetuses is displayed. The experiment was conducted as depicted in Figure 3A. Results of RNA Sequenom allelotyping experiments of selected imprinted transcripts listed to the left are shown using the color scale as in Figure 2. There were no statistically significant (*P* value <0.05) differences between ED and oil control, greater than 5%. The number (n) of G2 fetuses whose average values are shown per each ED is indicated. **(B)** Testing for transgenerational epigenetic inheritance of the aberrant imprinted expression. Average parental allele-specific transcription in different body parts/organs of three 13.5 dpc G3 fetuses is displayed. The experiment was performed as described in Figure 5A and displayed according to the color scale in Figure 2B. There were no statistically significant (*t*-test, *P* <0.05) differences between ED and oil control, greater than 5%. The number of G2 fetuses whose average values are shown per each ED is indicated. Additional file 3:
**Effect of EDs on allele-specific expression of imprinted genes in embryos derived from exposed primary oocytes.** The experiment was conducted as depicted in Figure 3B. Parental allele-specific transcription is displayed according to the color scale as in Figure 2. Results of RNA Sequenom allelotyping experiments of imprinted transcripts listed to the left are shown in different body parts/organs of the 13.5 dpc G2 embryo after ED or oil treatment of the G0 dam. Statistically significant (*P* <0.05) differences as compared to oil control greater than 5% and 10% are indicated by thin or bold rectangles, respectively. Note the strict undisturbed allele-specific transcription. Notice that there is no causative relationship between aberrant allele-specific DMR methylation (Figure 3D) and aberrant allele-specific transcription in this Figure. Additional file 4:
**Experimental designs for testing the effect of EDs on the establishment and maintenance of DNA methylation in the male germ line and for transgenerational inheritance of epigenetic aberrations.** **(A, B)** Experimental design to directly test the effect of ED exposure on paternal DNA methylation establishment in the offspring. G1 male offspring of FVB dam and OG2 father was exposed *in utero* at the time when paternal imprint establishment occurs in its prospermatogonia and MAT DMRs are protected from *de novo* DNA methylation. Exposure occurred daily from 12.5 dpc to 16.5 dpc by oral gavage to pregnant G0 dams with one of the three different EDs or vehicle control (oil). (A) Prospermatogonia were collected at 17.5 dpc for DNA methylation analysis from G1 fetuses by FACS sorting. In these prospermatognia, G1-specific DNA methylation is erased, and DNA methylation re-establishment is largely complete, resulting in reprogrammed G1 (G1R) pattern. (B) After reaching adulthood, G1R spermatozoa (developed from *in utero* exposed prospermatogonia) were also collected from G1 (FVBXOG2) males (green testicles). **(C, D)** Experimental design to test if perturbing DNA methylation establishment in prospermatogonia is transgenerationally inherited through the paternal germ line to an unexposed generation. G1 male offspring of FVB dam and FVB father was exposed *in utero* at the time when paternal imprint establishment occurs in its prospermatogonia and MAT DMRs are protected from *de novo* DNA methylation. Exposure occurred daily from 12.5 dpc to 16.5 dpc by oral gavage to pregnant G0 dams with one of the three different EDs or control oil vehicle. After reaching adulthood G1 male was mated with OG2 females and from G2 fetuses (derivative of exposed prospermatogonia, marked by three blue stars) prospermatogonia (never exposed to EDs) were collected for DNA methylation analysis by FACS sorting. These spermatogonia carried the reprogrammed G2 (G2R) DNA methylation pattern. (D) Some G2 (FVBXOG2) males were allowed to reach adulthood and were used for collecting G2R-type spermatozoa (developed from never exposed prospermatogonia). Additional file 5:
**MIRA-chip profile of imprinted DMRs in G1R and G2R prospermatogonia.** DNA methylation was mapped using MIRA-chip and custom Nimblegen arrays in prospermatogonia purified by FACS. The MIRA profile is depicted in the neighborhood of paternally (page 1) and maternally (page 2) methylated imprinted DMRs (black arrowheads) in biological triplicate samples for each treatment as indicated on the side. The DNA methylation signals of MIRA versus input DNA were plotted as -log10 *P* value scores ranging from 0 to 8.4 for gestational stage 17.5 dpc. The experiment was conducted as depicted in Figure 4. Note, that in prospermatogonia, default establishment of DNA methylation is undisturbed at paternally methylated DMRS and the protection from DNA methylation establishment is also undisturbed at maternally methylated imprinted DMRs. Additional file 6:
**No transgenerational epigenetic aberrations are detectable at imprinted DMRs after** ***in utero*** **ED exposure.** Samples (groups 5 to 8) were analyzed from the MIRA-chip experiment conducted using the custom imprinting arrays (Additional file 7). The average MIRA/input log2 ratios (n = 3 for MGC and n = 2 for sperm) were measured along known imprinted DMRs (column A), at the chromosomal locations indicated (columns B to D). The average values of two conditions were compared to detect changes along these DMRs with the cutoff values of ±5% and *P* <0.05. In the following columns the MIRA/input log2 ratios of each individual sample are also provided together with standard deviation values of the Student’s *t*-test. Additional file 7:
**List of MIRA-chip and Affymetrix samples.**
Additional file 8:
**Results of MIRA-chip experiments.** Prospermatogonia of G1 fetuses were treated with ED or oil control *in utero* as depicted in Additional file 4. DNA methylation was measured in purified G1R and G2R prospermatogonia (MGC) at 17.5 dpc and in adult spermatozoa by MIRA-chip. We used CpG-promoter or custom imprinting Nimblegen arrays. The analysis was conducted at four levels, as described in the legend to Table 3. In addition, at level 5, we compared the hits of level 1 to 4 analysis between the two types of arrays. Each peak (chromosomal coordinates are given in columns F to H) belongs to a level 1 test (column C) and may belong to a level 2 to 4 tests (column D) between two samples or two to four level 1 tests (column E). The MIRA intensities in each of the replicate and the average A and B samples are provided as log2 MIRA/input ratios (columns I to P). The differences in log2 MIRA/input ratios are given in column Q with color codes (red increase in A versus B, blue, decrease in A versus B) with *t*-test *P* values, *t*-test false discovery rates (FDR), Wilcoxon *P* values, and Wilcoxon FDRs. In case the MIRA peaks overlapped (+1 kb) with the upstream, intragenic, or downstream region of known transcripts, these were annotated using information on strand (±), start and end chromosomal coordinates, symbol, and accession number. Additional file 9:
**MIRA-chip profile at the top hits from a key study reporting transgenerationally inherited DNA methylation aberrations.** We treated prospermatogonia of G1 fetuses with VZ or oil control *in utero* as depicted in Additional file 4, and mapped DNA methylation using MIRA-chip and CpG-promoter Nimblegen arrays in purified G1R and G2R prospermatogonia (MGC) at 17.5 dpc and in adult spermatozoa. DNA methylation signals of MIRA versus input DNA were plotted as -log10 *P* values ranging from 0 to 8.3 for experimental and control replicate samples as indicated to the left. The regions represent the top hits from [43] where these regions exhibited the greatest decrease and increase (*Mro* and *Elf3*, respectively) in sperm of G3 adult males after *in utero* exposure of prospermatogonia inside G1 fetuses, and were considered examples for transgenerational epigenetic aberrations. Note the lack of change in G1R and G2R MGC and sperm at these locations (black triangles) and the complete lack of DNA methylation at the *Elf3* promoter at all times. Additional file 10:
**No transgenerational epigenetic aberrations are detectable at IAP elements after** ***in utero*** **ED exposure.** Samples (groups 5 to 8) were analyzed from the MIRA-chip experiment conducted using the custom imprinting arrays (Additional file 7). The average MIRA/input log2 ratios (n = 3 for MGCs and n = 2 for sperm) were measured along 1-kb-long flanking region to each IAP (column A), at the chromosomal locations indicated (columns B to D), where these flanks were unique sequences. Sheet 1 in the Excel file provides the complete dataset of the level 1 analysis. The average values of two conditions (average sample A versus average sample B) were compared along these regions to detect changes with the cutoff values of ±5% and *P* <0.05 (Student’s *t*-test). The MIRA/input log2 ratios of each individual sample are also provided together with standard deviation values of the Student’s *t*-test. Sheet 2 provides the result of the analysis where common changes were detected between MGC-sperm or between G1R and G2R samples. Sheet two provides the summary of common hits. In case the MIRA peaks overlapped (±10 kb) with the upstream, intragenic, or downstream region of known transcripts, these were annotated using information on strand (±), start and end chromosomal coordinates, symbol, and accession number. Note that there were very few immediate and persistent changes. These were small, often occurred in the opposite direction between generations, and rarely mapped to known transcripts. Additional file 11:
**Search for persistent epigenetic aberrations at IAP elements.** **(A)** Change in G1R MGC that persists into G2R MGC after *in utero* VZ exposure. DNA methylation was measured along each unique 1-kb-long IAP-flanking region in the MIRA-chip samples as shown at the top. Selected IAP-flank regions where common changes were detected in MGC samples between G1R MGC and G2R MGC are shown with chromosomal coordinates. The criteria for selection was the following: the change between G1R VZ versus G1R oil and also G2R VZ and G2R oil were at least ±5% with *P* <0.05 (Student’s *t*-test). The average MIRA/input log2 ratios (n = 3 for MGC and n = 2 for sperm) are depicted with red and blue flags in the range of -1.2 to +2.1. In case the MIRA peaks overlapped (±10 kb) with the upstream, intragenic, or downstream region of known transcripts, these were marked at the right using strand information (±). **(B)** Search for epigenetic aberrations at IAP elements in G1R MGC that persist into G1R sperm after *in utero* VZ exposure. The criteria for selection were the following: the change between MGC G1R VZ versus G1R oil and also between sperm G1R VZ and G2R oil was at least ±5% with *P* <0.05. **(C)** Search for epigenetic aberrations at IAP elements in G1R sperm that persist into G2R sperm after *in utero* VZ exposure. The criteria for selection was the following: the change between sperm G1R VZ versus G1R oil and also between sperm G2R VZ and G2R oil were at least ±5% with *P* <0.05. **(D)** Search for epigenetic aberrations at IAP elements in G1R MGC that persist into G2R MGC after *in utero* DEHP exposure. The criteria for selection were the following: the change between G1R DEHP versus G1R oil and also G2R DEHP and G2R oil were at least ±5% with *P* <0.05. **(E)** Search for epigenetic aberrations at IAP elements in G1R MGC that persist into G2 MGC after *in utero* BPA exposure. The criteria for selection were the following: the change between G1R BPA versus G1R oil and also G2R BPA and G2R oil were at least ±5% with *P* <0.05. Note that there were very few immediate and persistent changes. These were small and often occurred in the opposite direction between generations (bottom group). The full set of data and calculations are provided in Additional file 10. Additional file 12:
**Common changes of transcription in G1R and G2R prospermatogonia.** **(A)** Common changes after DEHP treatment. **(B)** Common changes after VZ treatment. Full results of the common hits tabulated in Table 4 is shown. Common changes were detected between the unique transcripts that change in G1R and G2R samples for the same treatment using 1.05-fold difference and *P* <0.05 cutoff values. We marked those that changed in the same direction. Additional file 13:
**List of primers used for Sequenom allelotyping.** Primers are listed for Sequenom experiments. The first PCR primer (1st-PCRP) and the second PCR primer (2nd-PCRP) were used in the amplification. UEP_SEQ primers were used in mass spectrometry after incorporating one or the other allele-specific nucleotide by primer extension. The DMR18-plex was used for DNA methylation analysis at DMR regions. The imprinted expression multiplexes were used for allele-specific transcription analysis at imprinted genes.

## Abbreviations

BPA: Bisphenol A
DEHP: Di-(2-ethylhexyl) phthalate
DMR: Differentially methylated region
ED: Endocrine-disrupting chemical
FDR: False discovery rate
FGC: Female germ cell
FSC: Female somatic cell
G1R: Generation 1–reprogrammed
G2R: Generation 2-reprogrammed
IAP: Intracisternal A-particle element
MAT: Maternally methylated
MGC: Male germ cell
MIRA: Methylated CpG island recovery assay
MSC: Male somatic cell
PAT: Paternally methylated
PCA: Principal component analysis
PGC: Primordial germ cells
TGI: Transgenerational inheritance
VZ: Vinclozolin
