# Supplementary figures and images for: Deleterious effects of endocrine disruptors are corrected in the mammalian germline by epigenome reprogramming
Source: Genome Biol. 2015 Mar 27;16(1):59. doi: 10.1186/s13059-015-0619-z (PMC4376074; doi:10.1186/s13059-015-0619-z)

A

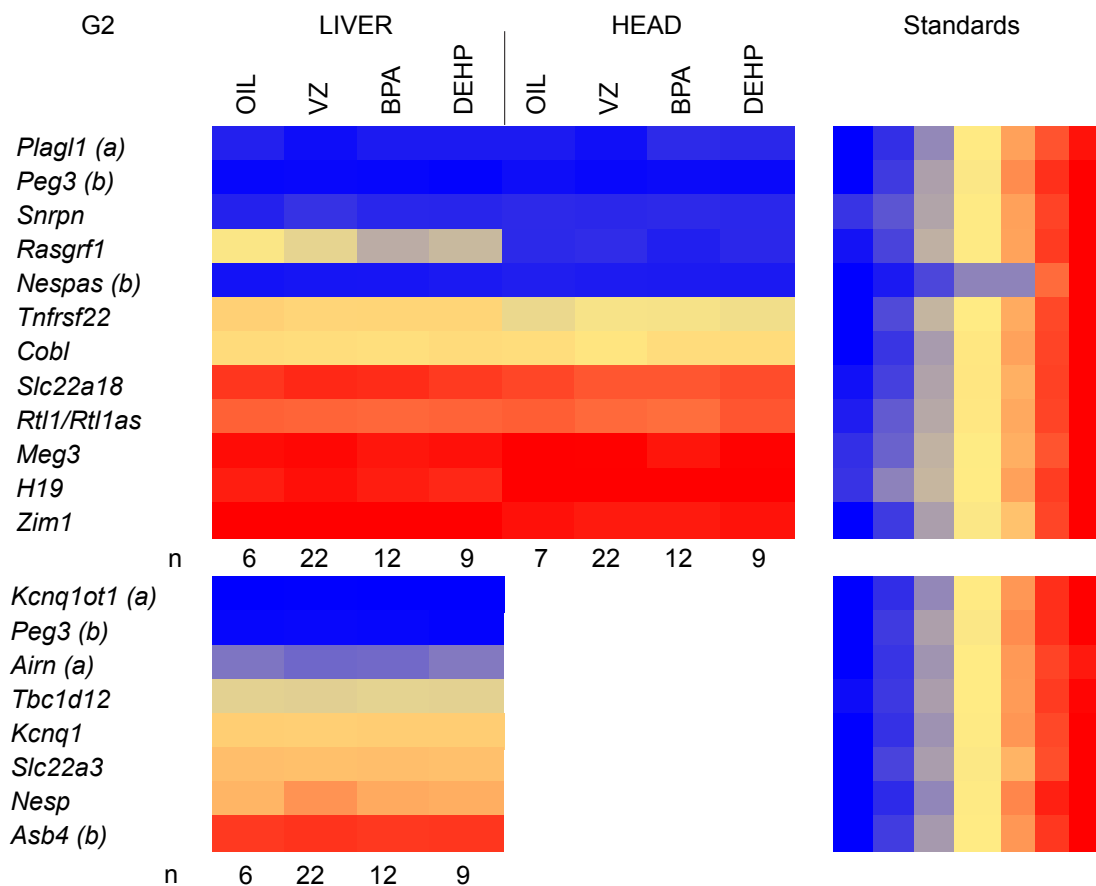

B

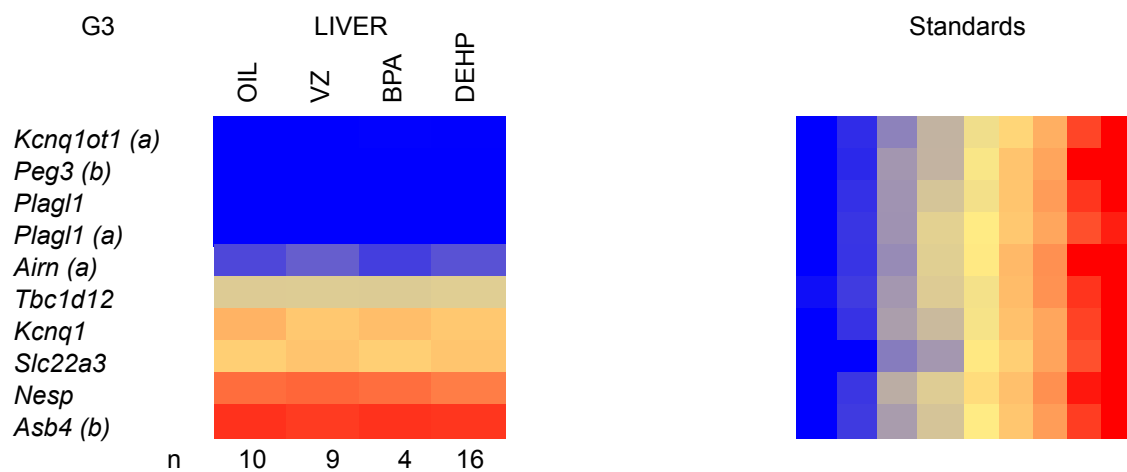

Supplement: Additional file 2: — Analysis of larger number of fetuses. (A) Effect of EDs on allele-specific expression of imprinted genes in fetuses derived from exposed prospermatogonia. Average parental allele-specific transcription in different body parts/organs of three 13.5 dpc G2 fetuses is displayed. The experiment was conducted as depicted in Figure 3A. Results of RNA Sequenom allelotyping experiments of selected imprinted transcripts listed to the left are shown using the color scale as in Figure 2. There were no statistically significant (P value <0.05) differences between ED and oil control, greater than 5%. The number (n) of G2 fetuses whose average values are shown per each ED is indicated. (B) Testing for transgenerational epigenetic inheritance of the aberrant imprinted expression. Average parental allele-specific transcription in different body parts/organs of three 13.5 dpc G3 fetuses is displayed. The experiment was performed as described in Figure 5A and displayed according to the color scale in Figure 2B. There were no statistically significant (t-test, P <0.05) differences between ED and oil control, greater than 5%. The number of G2 fetuses whose average values are shown per each ED is indicated. [file 13059_2015_619_MOESM2_ESM.pdf]

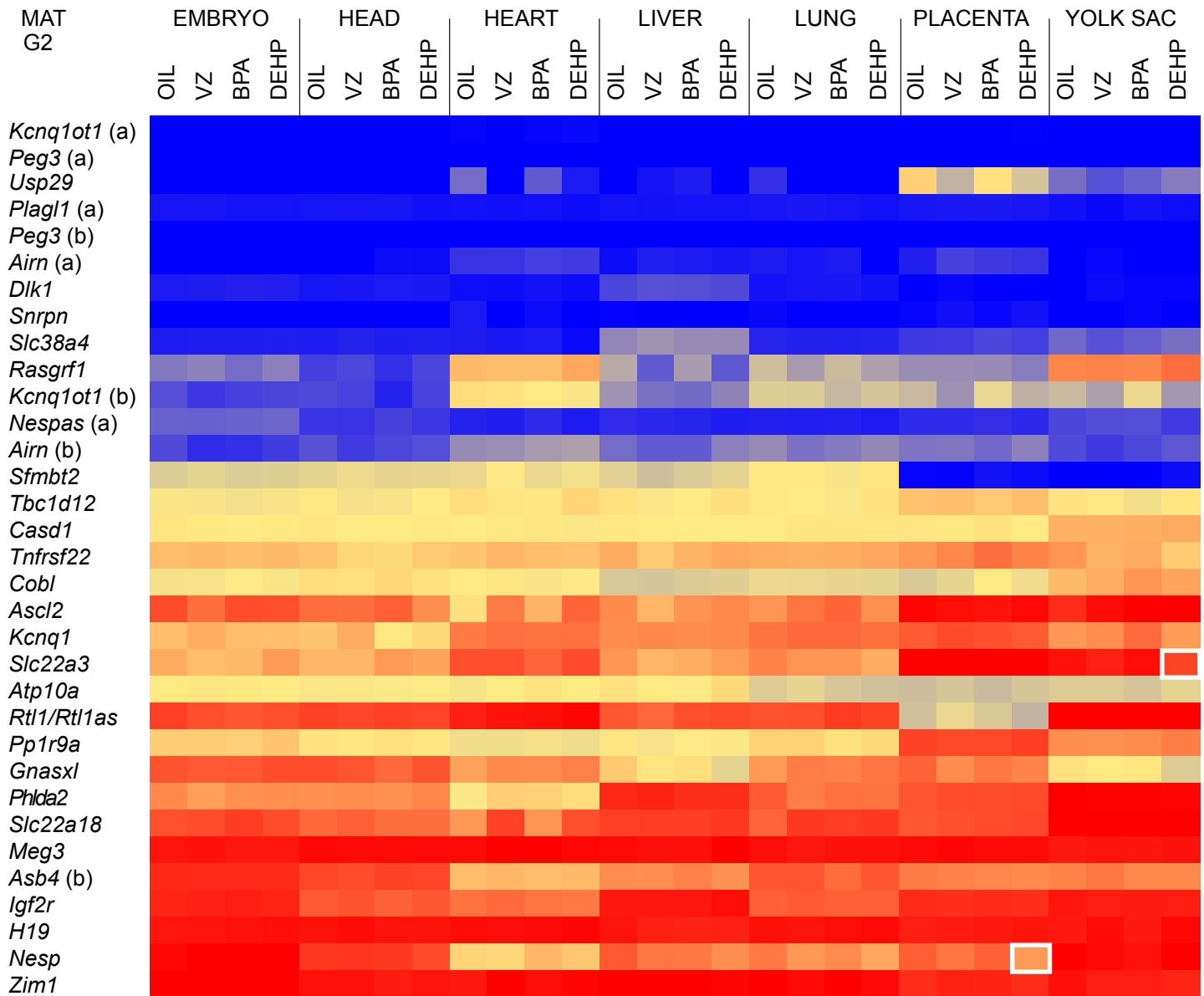

Supplement: Additional file 3: — Effect of EDs on allele-specific expression of imprinted genes in embryos derived from exposed primary oocytes. The experiment was conducted as depicted in Figure 3B. Parental allele-specific transcription is displayed according to the color scale as in Figure 2. Results of RNA Sequenom allelotyping experiments of imprinted transcripts listed to the left are shown in different body parts/organs of the 13.5 dpc G2 embryo after ED or oil treatment of the G0 dam. Statistically significant (P <0.05) differences as compared to oil control greater than 5% and 10% are indicated by thin or bold rectangles, respectively. Note the strict undisturbed allele-specific transcription. Notice that there is no causative relationship between aberrant allele-specific DMR methylation (Figure 3D) and aberrant allele-specific transcription in this Figure. [file 13059_2015_619_MOESM3_ESM.pdf]

A

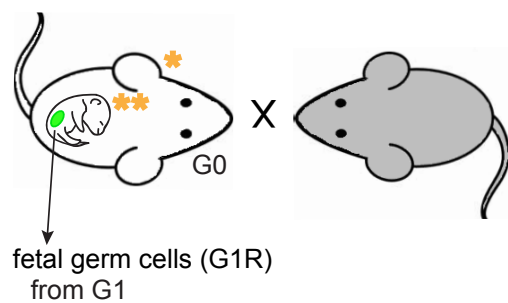

C

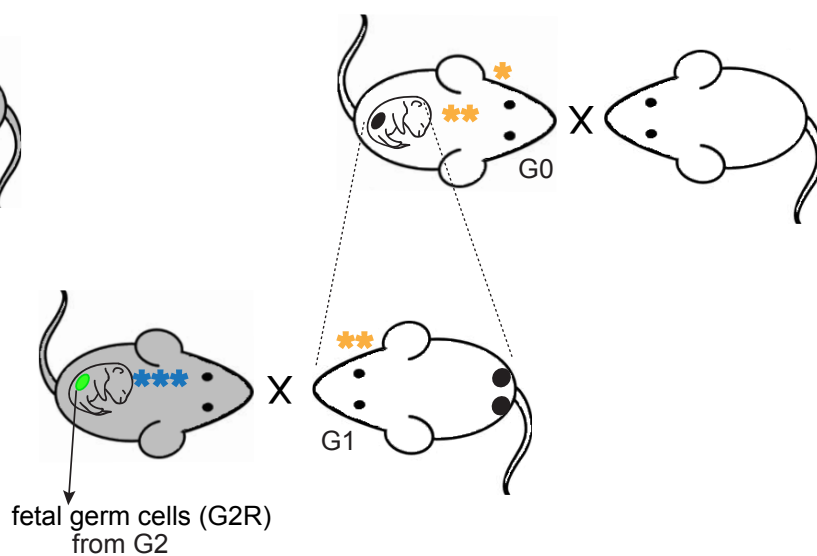

B

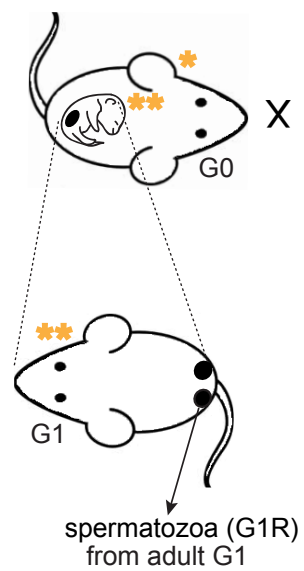

D

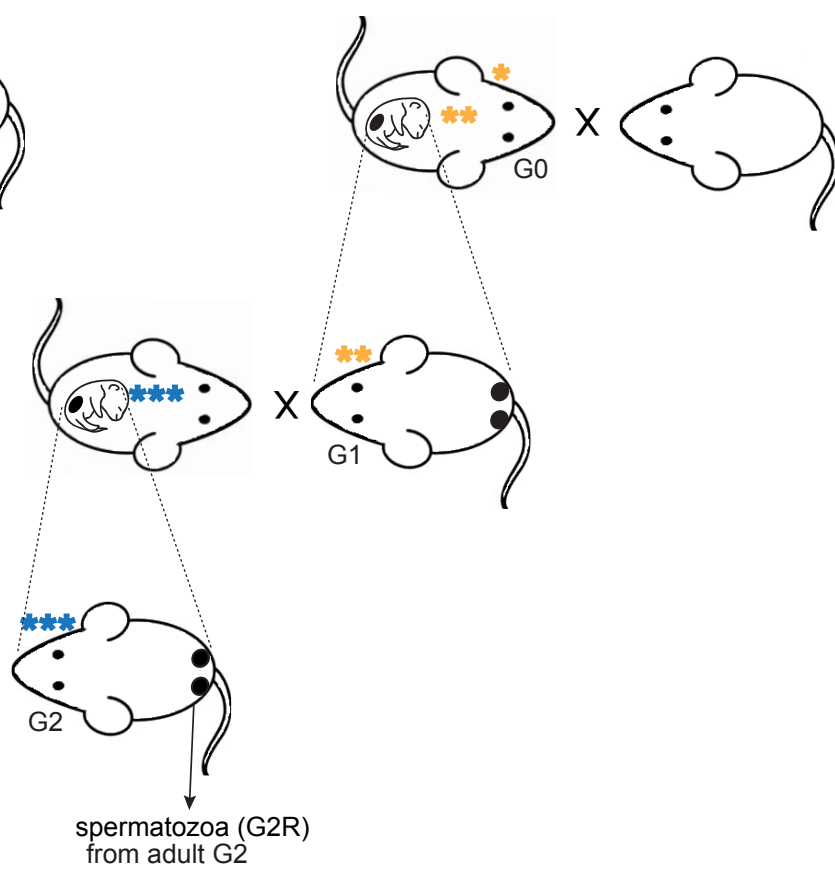

Supplement: Additional file 4: — Experimental designs for testing the effect of EDs on the establishment and maintenance of DNA methylation in the male germ line and for transgenerational inheritance of epigenetic aberrations. (A, B) Experimental design to directly test the effect of ED exposure on paternal DNA methylation establishment in the offspring. G1 male offspring of FVB dam and OG2 father was exposed in utero at the time when paternal imprint establishment occurs in its prospermatogonia and MAT DMRs are protected from de novo DNA methylation. Exposure occurred daily from 12.5 dpc to 16.5 dpc by oral gavage to pregnant G0 dams with one of the three different EDs or vehicle control (oil). (A) Prospermatogonia were collected at 17.5 dpc for DNA methylation analysis from G1 fetuses by FACS sorting. In these prospermatognia, G1-specific DNA methylation is erased, and DNA methylation re-establishment is largely complete, resulting in reprogrammed G1 (G1R) pattern. (B) After reaching adulthood, G1R spermatozoa (developed from in utero exposed prospermatogonia) were also collected from G1 (FVBXOG2) males (green testicles). (C, D) Experimental design to test if perturbing DNA methylation establishment in prospermatogonia is transgenerationally inherited through the paternal germ line to an unexposed generation. G1 male offspring of FVB dam and FVB father was exposed in utero at the time when paternal imprint establishment occurs in its prospermatogonia and MAT DMRs are protected from de novo DNA methylation. Exposure occurred daily from 12.5 dpc to 16.5 dpc by oral gavage to pregnant G0 dams with one of the three different EDs or control oil vehicle. After reaching adulthood G1 male was mated with OG2 females and from G2 fetuses (derivative of exposed prospermatogonia, marked by three blue stars) prospermatogonia (never exposed to EDs) were collected for DNA methylation analysis by FACS sorting. These spermatogonia carried the reprogrammed G2 (G2R) DNA methylation pattern. (D) Some G2 (FVBXOG2) mal [file 13059_2015_619_MOESM4_ESM.pdf]

**A**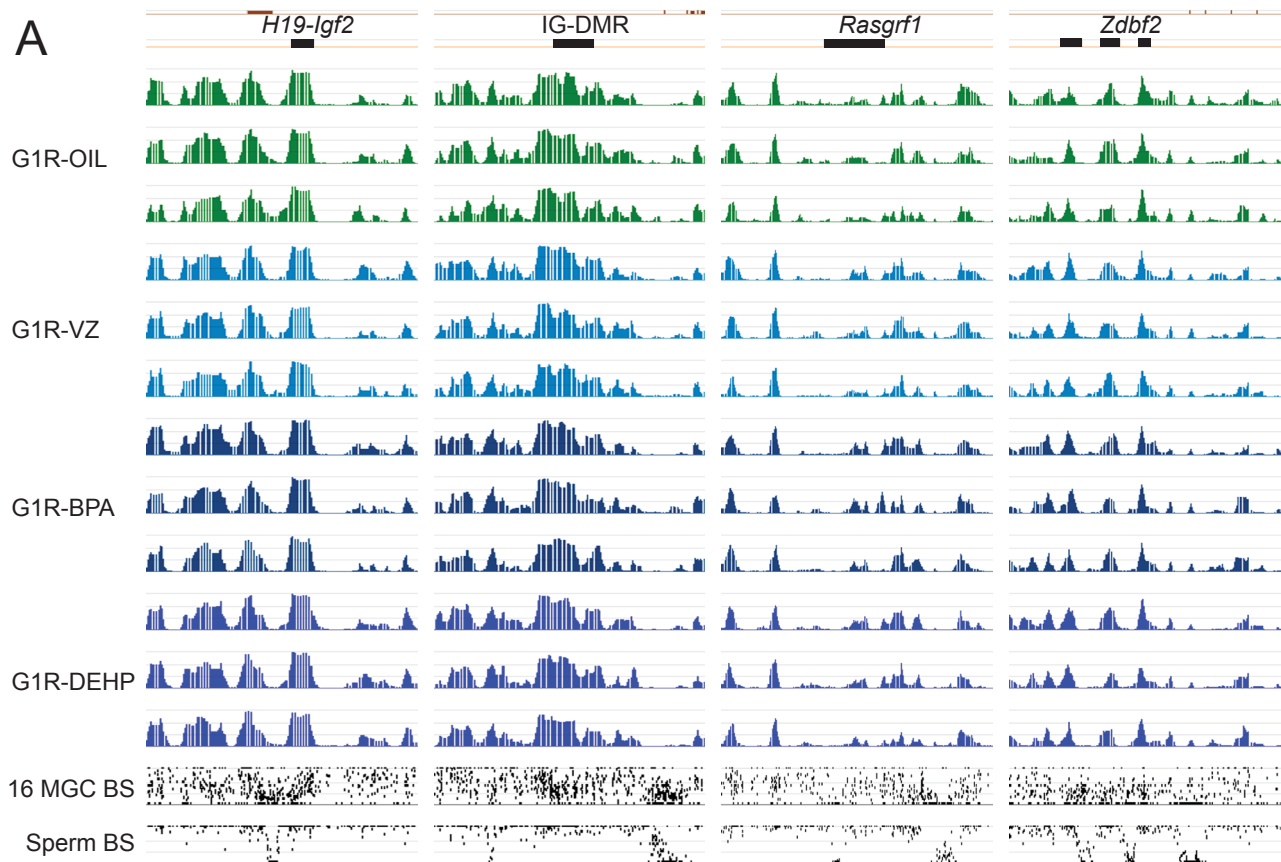**B**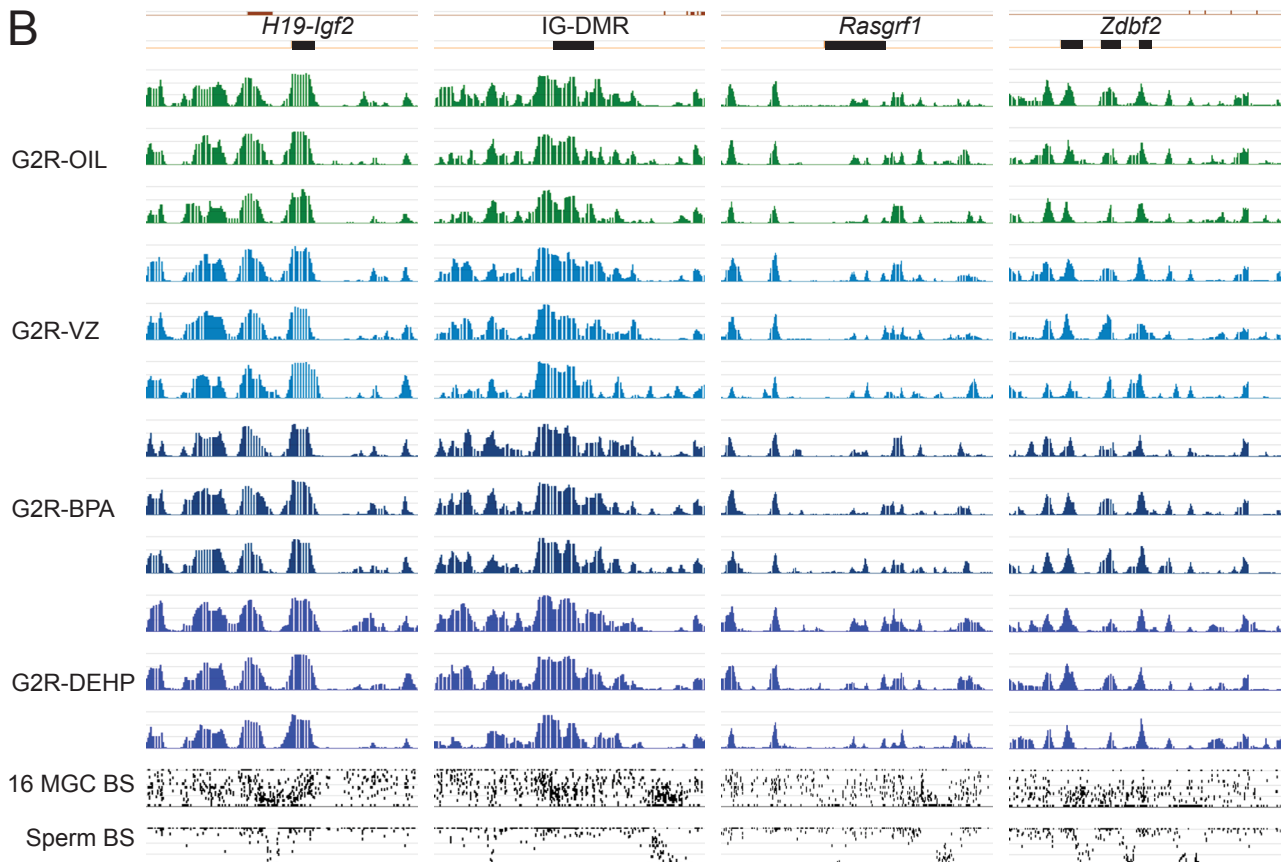

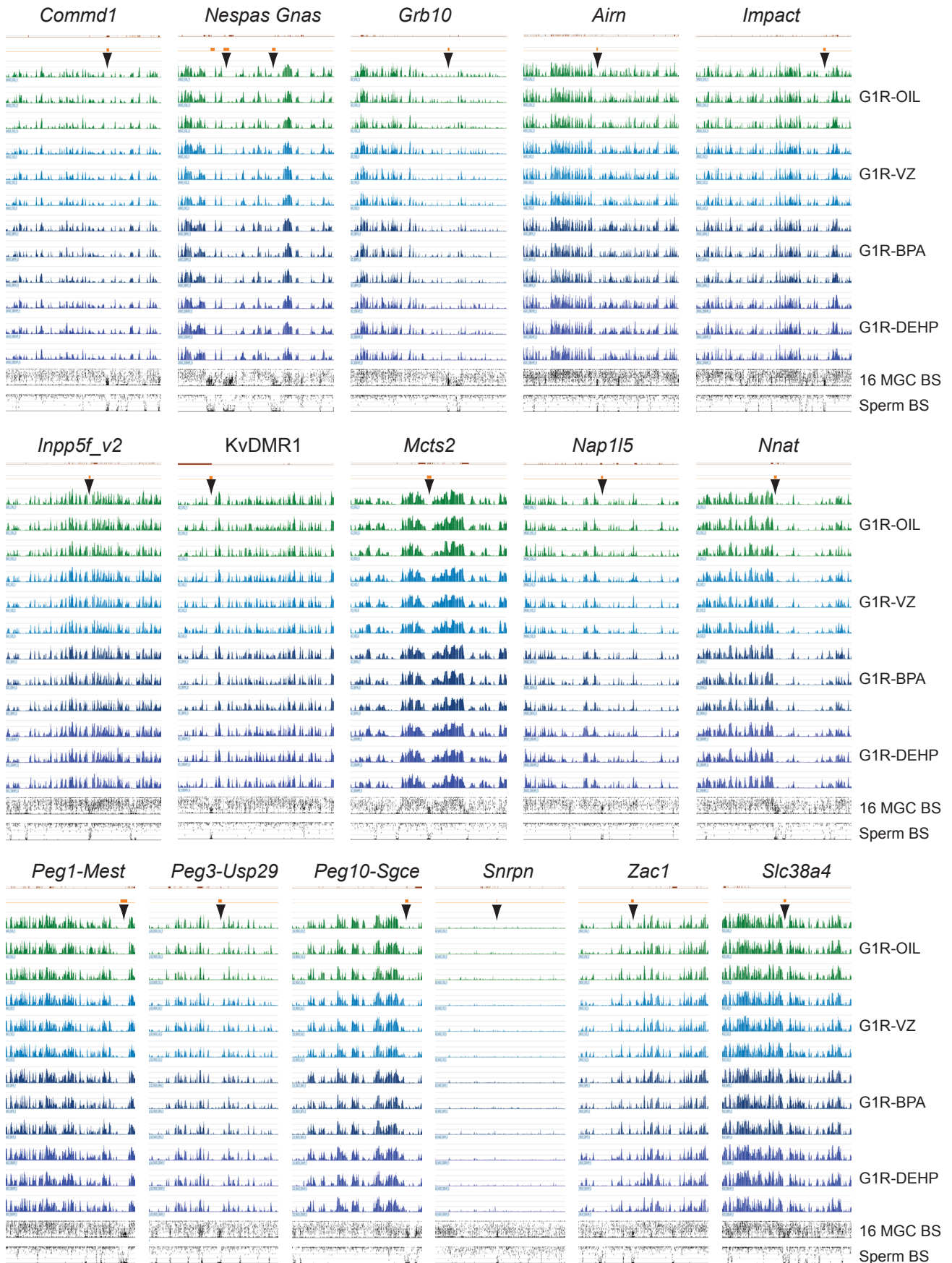

Supplement: Additional file 5: — MIRA-chip profile of imprinted DMRs in G1R and G2R prospermatogonia. DNA methylation was mapped using MIRA-chip and custom Nimblegen arrays in prospermatogonia purified by FACS. The MIRA profile is depicted in the neighborhood of paternally (page 1) and maternally (page 2) methylated imprinted DMRs (black arrowheads) in biological triplicate samples for each treatment as indicated on the side. The DNA methylation signals of MIRA versus input DNA were plotted as -log10 P value scores ranging from 0 to 8.4 for gestational stage 17.5 dpc. The experiment was conducted as depicted in Figure 4. Note, that in prospermatogonia, default establishment of DNA methylation is undisturbed at paternally methylated DMRS and the protection from DNA methylation establishment is also undisturbed at maternally methylated imprinted DMRs. [file 13059_2015_619_MOESM5_ESM.pdf]

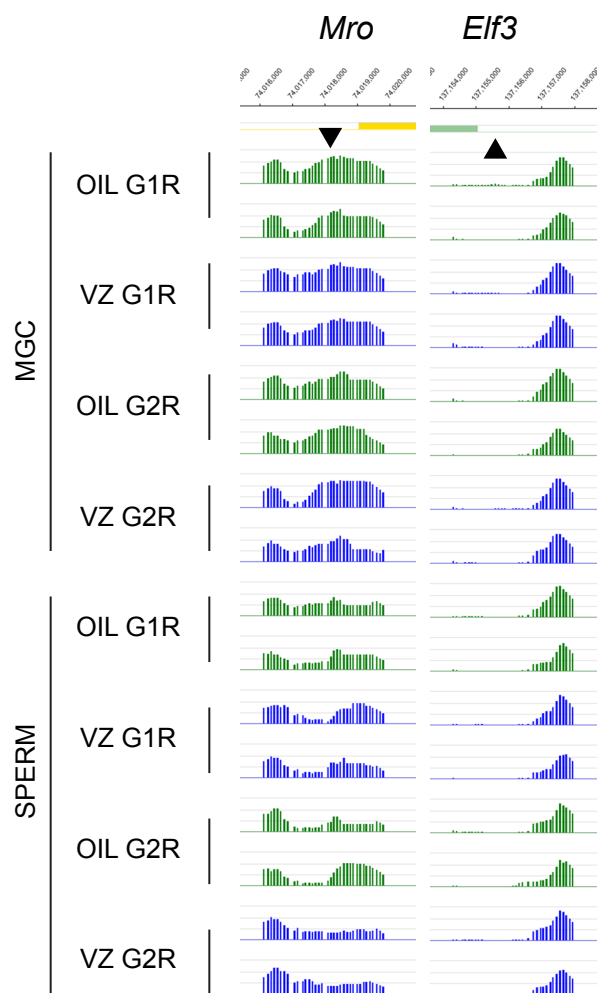

Supplement: Additional file 9: — MIRA-chip profile at the top hits from a key study reporting transgenerationally inherited DNA methylation aberrations. We treated prospermatogonia of G1 fetuses with VZ or oil control in utero as depicted in Additional file 4, and mapped DNA methylation using MIRA-chip and CpG-promoter Nimblegen arrays in purified G1R and G2R prospermatogonia (MGC) at 17.5 dpc and in adult spermatozoa. DNA methylation signals of MIRA versus input DNA were plotted as -log10 P values ranging from 0 to 8.3 for experimental and control replicate samples as indicated to the left. The regions represent the top hits from [43] where these regions exhibited the greatest decrease and increase (Mro and Elf3, respectively) in sperm of G3 adult males after in utero exposure of prospermatogonia inside G1 fetuses, and were considered examples for transgenerational epigenetic aberrations. Note the lack of change in G1R and G2R MGC and sperm at these locations (black triangles) and the complete lack of DNA methylation at the Elf3 promoter at all times. [file 13059_2015_619_MOESM9_ESM.pdf]

## VZ MGC G1R-G2R

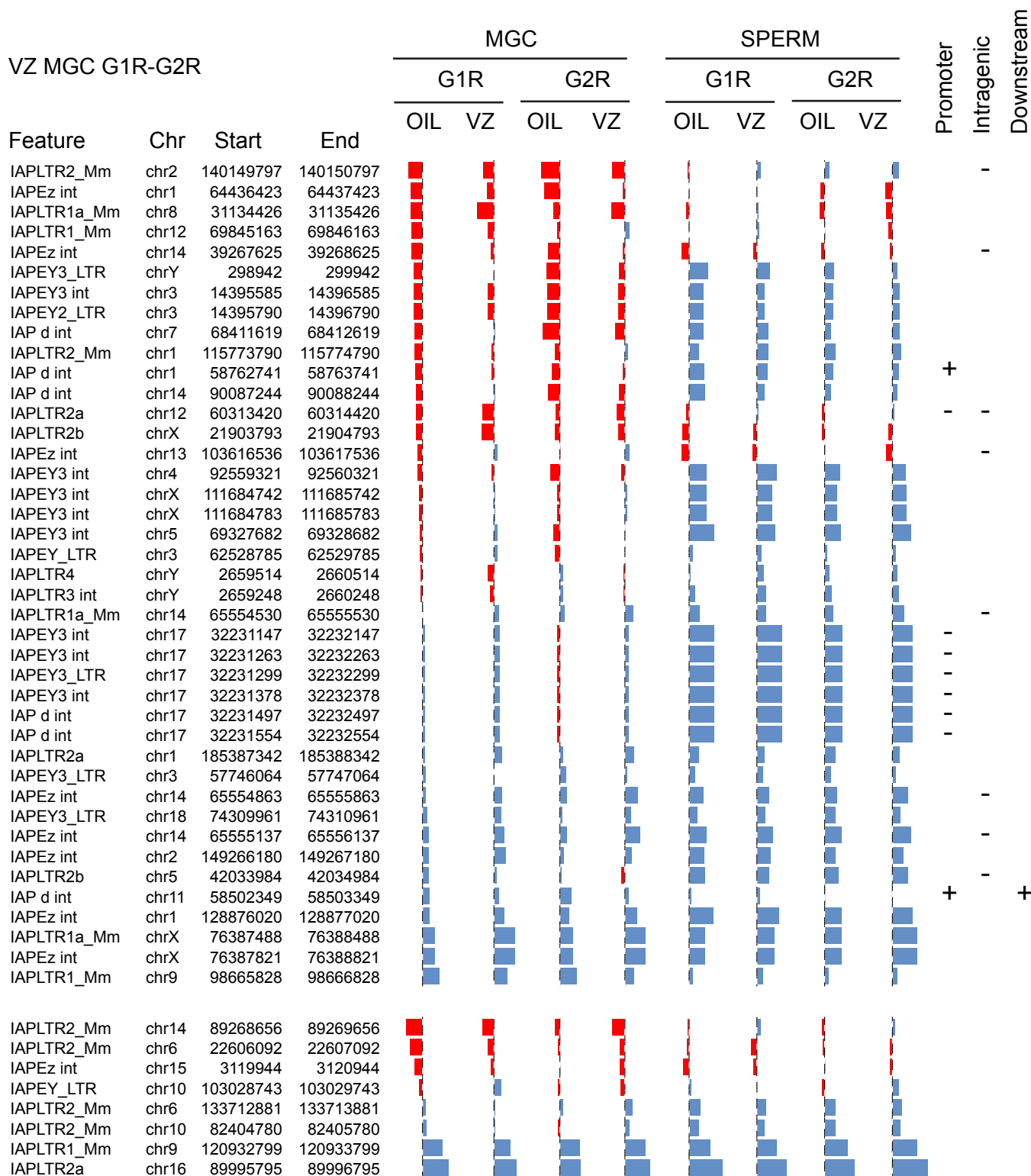

log2 R -1.2 ■ 2.1

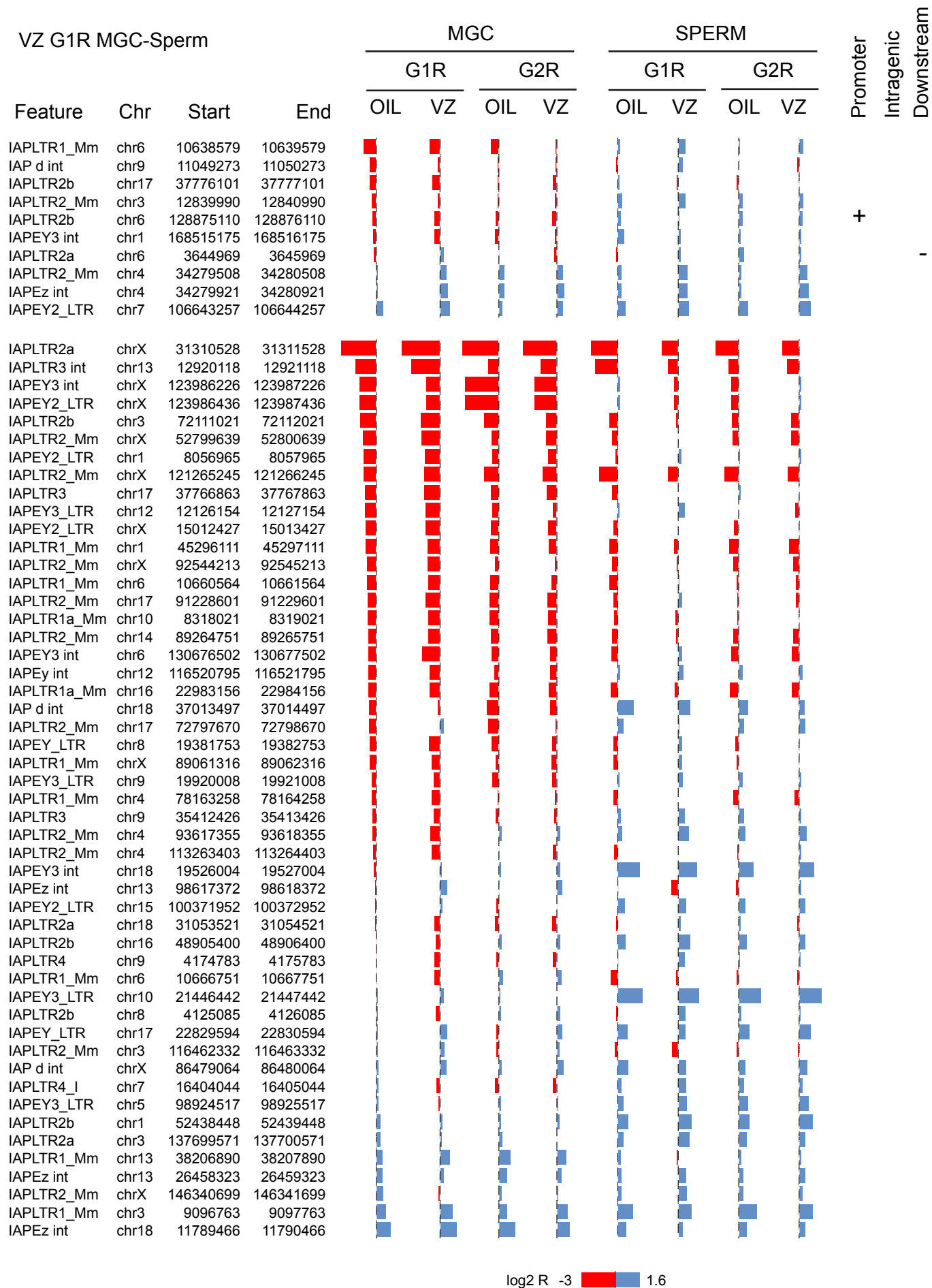

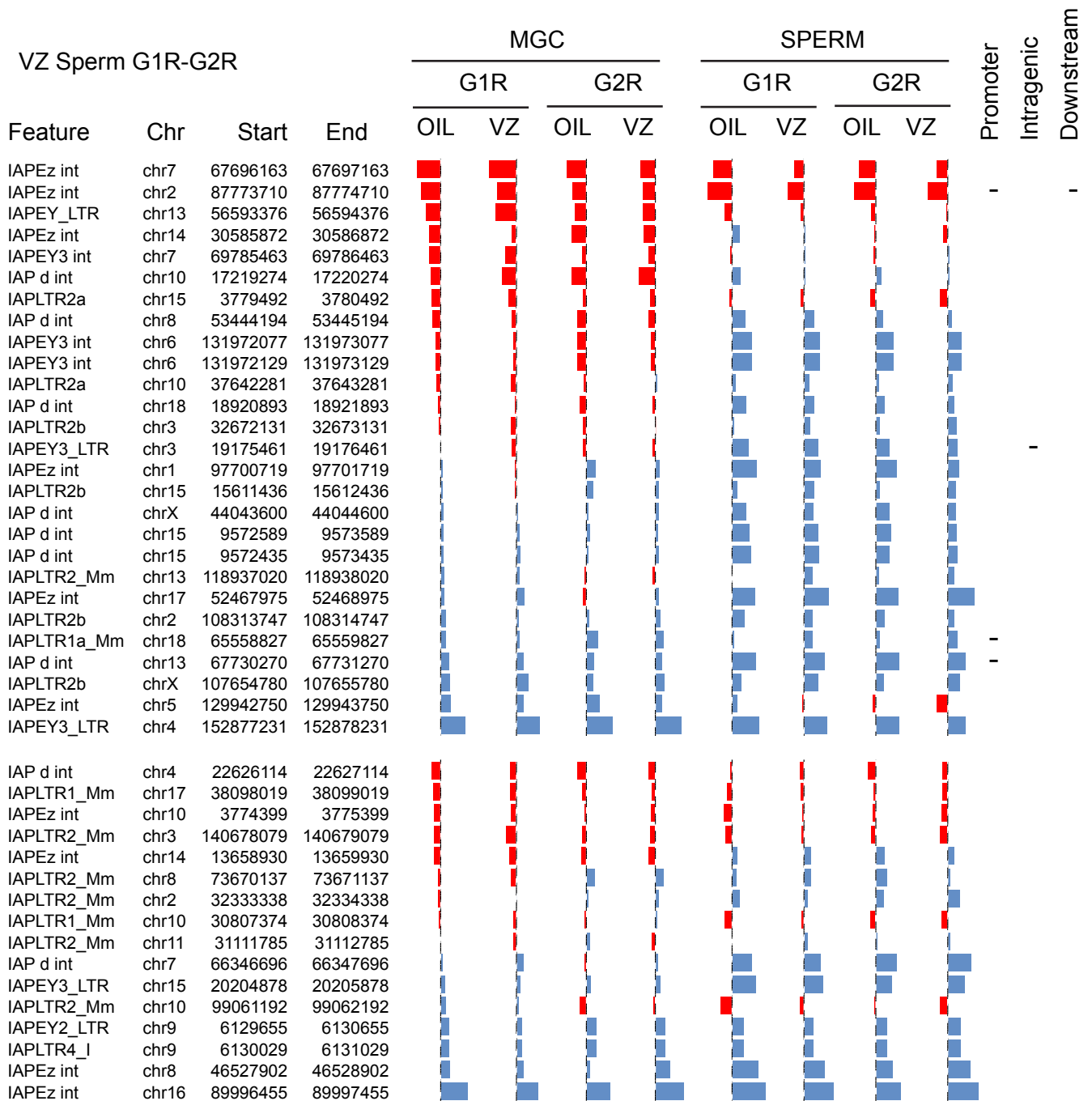

log2 R -1.8 ■ 1.8

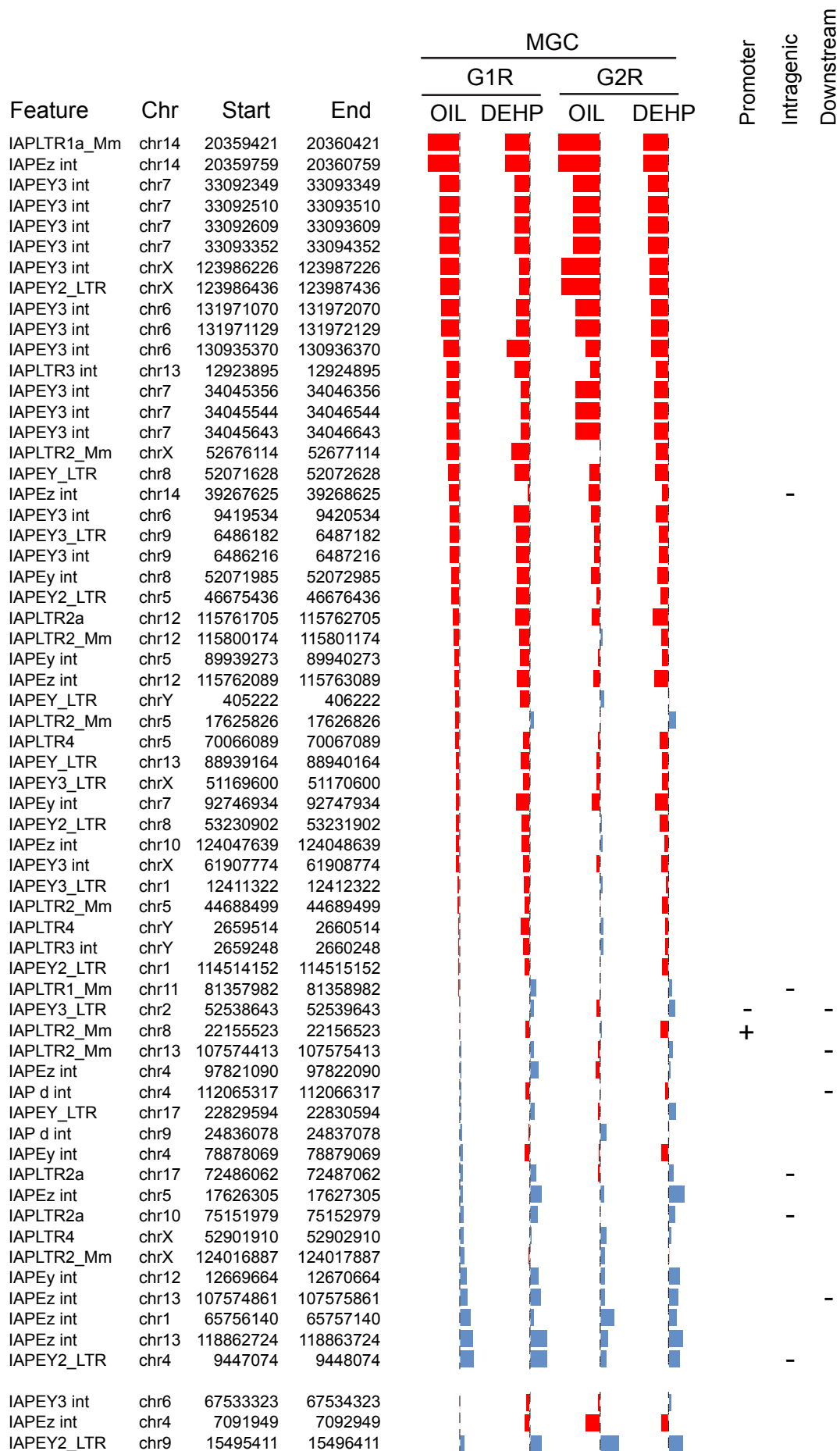

log2 R -2 ■ 1

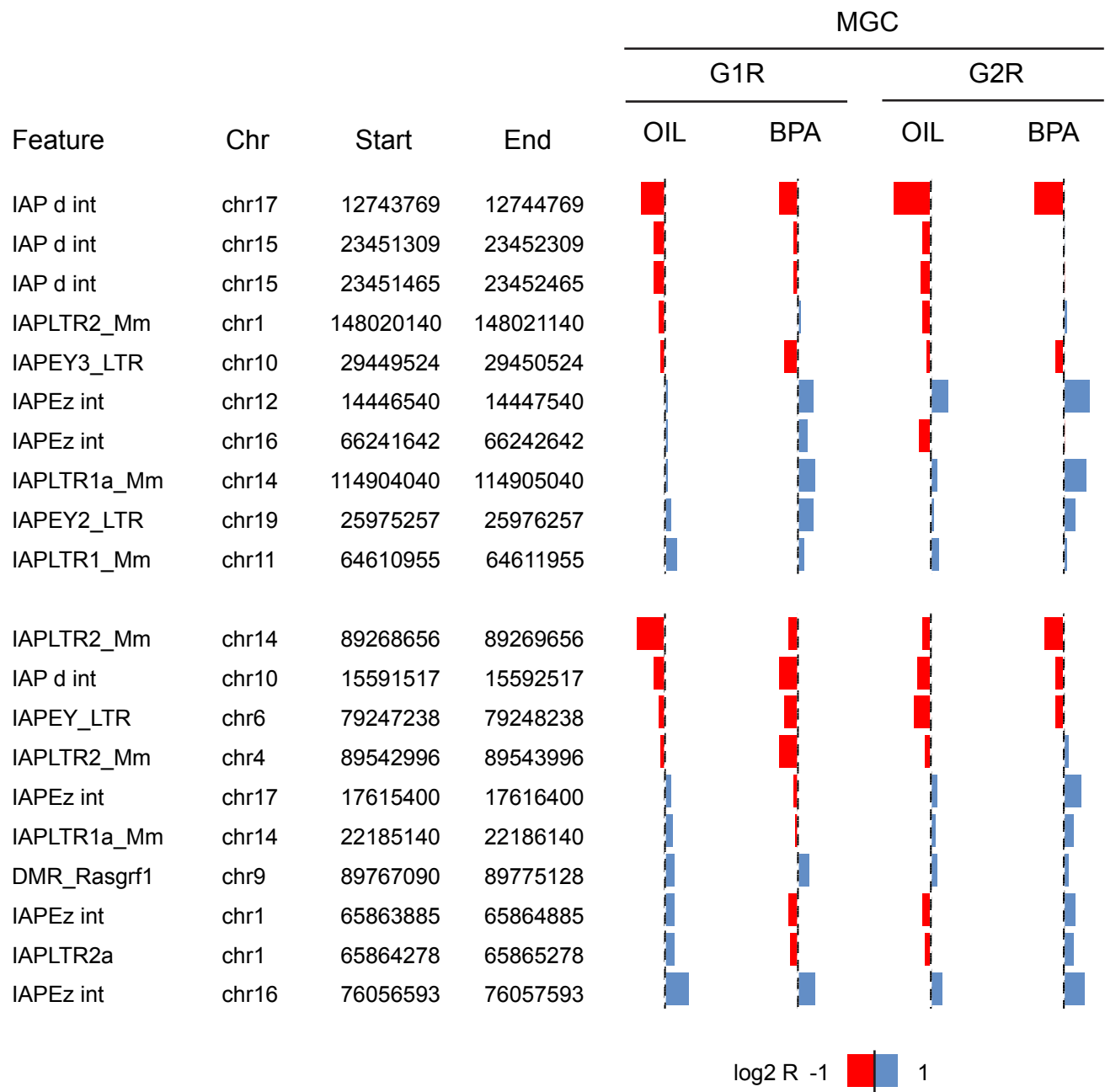

Supplement: Additional file 11: — Search for persistent epigenetic aberrations at IAP elements. (A) Change in G1R MGC that persists into G2R MGC after in utero VZ exposure. DNA methylation was measured along each unique 1-kb-long IAP-flanking region in the MIRA-chip samples as shown at the top. Selected IAP-flank regions where common changes were detected in MGC samples between G1R MGC and G2R MGC are shown with chromosomal coordinates. The criteria for selection was the following: the change between G1R VZ versus G1R oil and also G2R VZ and G2R oil were at least ±5% with P <0.05 (Student’s t-test). The average MIRA/input log2 ratios (n = 3 for MGC and n = 2 for sperm) are depicted with red and blue flags in the range of -1.2 to +2.1. In case the MIRA peaks overlapped (±10 kb) with the upstream, intragenic, or downstream region of known transcripts, these were marked at the right using strand information (±). (B) Search for epigenetic aberrations at IAP elements in G1R MGC that persist into G1R sperm after in utero VZ exposure. The criteria for selection were the following: the change between MGC G1R VZ versus G1R oil and also between sperm G1R VZ and G2R oil was at least ±5% with P <0.05. (C) Search for epigenetic aberrations at IAP elements in G1R sperm that persist into G2R sperm after in utero VZ exposure. The criteria for selection was the following: the change between sperm G1R VZ versus G1R oil and also between sperm G2R VZ and G2R oil were at least ±5% with P <0.05. (D) Search for epigenetic aberrations at IAP elements in G1R MGC that persist into G2R MGC after in utero DEHP exposure. The criteria for selection were the following: the change between G1R DEHP versus G1R oil and also G2R DEHP and G2R oil were at least ±5% with P <0.05. (E) Search for epigenetic aberrations at IAP elements in G1R MGC that persist into G2 MGC after in utero BPA exposure. The criteria for selection were the following: the change between G1R BPA versus G1R oil and also G2R BPA and G2R oil were at least ±5% with P [file 13059_2015_619_MOESM11_ESM.pdf]
